# *Drosophila melanogaster* Acetyl-CoA-Carboxylase Sustains a Fatty Acid–Dependent Remote Signal to Waterproof the Respiratory System

**DOI:** 10.1371/journal.pgen.1002925

**Published:** 2012-08-30

**Authors:** Jean-Philippe Parvy, Laura Napal, Thomas Rubin, Mickael Poidevin, Laurent Perrin, Claude Wicker-Thomas, Jacques Montagne

**Affiliations:** 1 CNRS, Centre de Génétique Moléculaire, UPR 3404, Gif-sur-Yvette, France; 2 Université Pierre et Marie Curie- Paris 6, Paris, France; 3 Université Paris-Sud 11, Orsay, France; 4 IBDML, Université de la Méditerranée, Marseille, France; 5 CNRS, LEGS, UPR 9034, Gif-sur-Yvette, France; Northwestern University, United States of America

## Abstract

Fatty acid (FA) metabolism plays a central role in body homeostasis and related diseases. Thus, FA metabolic enzymes are attractive targets for drug therapy. Mouse studies on Acetyl-coenzymeA-carboxylase (ACC), the rate-limiting enzyme for FA synthesis, have highlighted its homeostatic role in liver and adipose tissue. We took advantage of the powerful genetics of *Drosophila melanogaster* to investigate the role of the unique *Drosophila* ACC homologue in the fat body and the oenocytes. The fat body accomplishes hepatic and storage functions, whereas the oenocytes are proposed to produce the cuticular lipids and to contribute to the hepatic function. RNA–interfering disruption of ACC in the fat body does not affect viability but does result in a dramatic reduction in triglyceride storage and a concurrent increase in glycogen accumulation. These metabolic perturbations further highlight the role of triglyceride and glycogen storage in controlling circulatory sugar levels, thereby validating *Drosophila* as a relevant model to explore the tissue-specific function of FA metabolic enzymes. In contrast, ACC disruption in the oenocytes through RNA–interference or tissue-targeted mutation induces lethality, as does oenocyte ablation. Surprisingly, this lethality is associated with a failure in the watertightness of the spiracles—the organs controlling the entry of air into the trachea. At the cellular level, we have observed that, in defective spiracles, lipids fail to transfer from the spiracular gland to the point of air entry. This phenotype is caused by disrupted synthesis of a putative very-long-chain-FA (VLCFA) within the oenocytes, which ultimately results in a lethal anoxic issue. Preventing liquid entry into respiratory systems is a universal issue for air-breathing animals. Here, we have shown that, in *Drosophila*, this process is controlled by a putative VLCFA produced within the oenocytes.

## Author Summary

Fatty acid homeostasis is deregulated in several human diseases, including obesity, diabetes, and most cancers. Therefore, the enzymes that catalyze the reactions of fatty acid metabolism constitute attractive targets for drug therapy. However, the development of novel inhibitors requires extensive analysis of the organ-specific functions of the targeted enzyme. Given the availability of genetic tools, the fruit fly *Drosophila* is an appropriate model system to investigate the physiological and developmental roles of metabolic enzymes. Here we studied a *Drosophila* homologue of a rate-limiting enzyme for fatty acid synthesis. We have shown that this enzyme is necessary to control the storage of lipids in the fat tissue, validating our system to study fatty acid metabolism. We further observed that this enzyme is essential in the oenocytes, a group of cells proposed to contribute to the hepatic function and to the formation of the cuticle. Furthermore, we have shown that a putative fatty acid produced in these cells is required to control, at a distance, the watertightness of the respiratory system. In summary, our study identifies a novel fatty acid-mediated signal necessary to prevent liquid accumulation in the respiratory system, a critical issue for all air-breathing animals.

## Introduction

Fatty acids (FAs) are the most abundant energy stores in animals and are essential precursors of membrane components and signaling molecules [1], [2]. FA metabolism is also tightly linked to human diseases. Increased levels of circulatory FAs and their accumulation as triglycerides (TGs) in adipocytes constitute a critical step in the development of obesity and type 2 diabetes [3]. Hyperactive *de novo* FA synthesis is characteristic of most cancer cells and the underlying enzymes are therefore attractive targets for drug therapy [4], [5]. It is crucial, then, to assess the role of FA metabolic enzymes at the level of an entire organism and to determine how the various tissue-specific activities cooperate to maintain body homeostasis.

In mammals, the liver is central to the coordination of FA synthesis and oxidation in response to dietary status [6], [7]. Nutrients crossing the gut epithelium enter into blood circulation to reach the liver. Some glucose enters into the hepatocytes to be metabolized as glycogen or FAs. Most of these FAs are transported as TGs to be stored in adipocytes. Fasting induces the remobilization of TGs from adipose tissue to the liver [8], where they are oxidized to produce ketone bodies as an energy source for peripheral tissues. This remobilization provokes the accumulation of lipid droplets (LDs) in the hepatocytes, a phenomenon called fatty liver that is also a pathological symptom of metabolic syndrome [9].

In *Drosophila melanogaster*, the fat body (FB) performs adipose- and hepatic-like functions [10]. Most of the nutrients assimilated are transferred into the FB to be metabolized and stored as TGs [11]. Glucose is also stored as glycogen in the FB of adult flies [12] and in the body wall muscles of feeding larvae [13]. The oenocytes have also been shown to accomplish a hepatic-like function and to regulate lipid metabolism [14]. Oenocytes are groups of cells located under the tegument on both sides of each abdominal segment. In several insect species, the oenocytes are tightly connected with the abdominal spiracles, the organs controlling the entry of air into the respiratory system [15]. In contrast to insects exhibiting metameric spiracles, *Drosophila* larvae develop only two pairs of spiracles, located at the posterior and anterior ends [16]. Upon fasting, the oenocytes accumulate large amounts of LDs as in fast-induced fatty liver [14]. Oenocytes are also proposed to regulate growth, since their genetic ablation induces a growth arrest and animal death during larval development. However, the deregulated processes that eventually result in animal death following oenocyte ablation remain uncharacterized.

The basic path of *de novo* FA synthesis requires two major enzymatic steps. First, Acetyl-CoA-carboxylase (ACC) catalyzes the carboxylation of acetyl-CoA to produce malonyl-CoA [17]. Next, fatty acid synthase (FAS) sequentially condensates several malonyl-CoA molecules with one acetyl-CoA primer to build up long chain FAs (LCFAs); the most abundant form of LCFA is palmitate (C16) [18]. Synthesis of very-long-chain-FAs (VLCFAs) is catalyzed by a multi-enzymatic complex that also utilizes malonyl-CoA [19]. Unlike FAS, which is found in the cytosol, this complex is bound to the endoplasmic reticulum and is made up of four enzyme subunits that further elongate a fatty-acyl-CoA substrate. Incorporation of malonyl-CoA is first catalyzed by a member of the elongase family, whose various gene products differ from one another in their tissue-specific expression and substrate specificity. The subsequent steps are successively catalyzed by a 3-Keto-acyl-CoA-reductase (KAR), a 3-Hydroxy-acyl-CoA-dehydratase (HADC) and a Trans-enoyl-CoA-reductase (TER) [20].

In mice, ACC is encoded by two distinct genes *ACC1* and *ACC2*. *ACC1* is mainly expressed in lipogenic tissues to produce malonyl-CoA as a precursor to FA synthesis [21]. *ACC2* is expressed in oxidative tissues to produce a pool of malonyl-CoA [22] that is assumed to control β-oxidation by inhibiting the activity of CPTI (Carnitine palmitoyltransferase I) and COT (Carnitine octanoyltransferase); these two enzymes transfer the fatty acyl-CoA substrate into mitochondria and peroxysomes, respectively [23]. Only *ACC1* is essential; knockout mice die at embryonic day E8,5 for a yet unknown reason [21]. *ACC2* knockout mice are viable, but they continuously oxidize their FAs [22]. Conditional knockout of *ACC1* does not affect viability when targeted to the liver [24] or the adipose tissue, although the latter results in a skeletal growth defect [25]. Knocking out the single mouse *FAS* gene leads to pre-implantation lethality [26]. Conditional *FAS* knockout targeted to the liver (*FASKOL*) does not affect viability [27]. *FASKOL* mice have lower glucose, insulin and cholesterol levels. Surprisingly, these mice have fatty liver when fed on a fat-free diet, suggesting that FAs may be synthesized in other tissues and subsequently remobilized to the liver. Together, these results suggest that *de novo* FA synthesis is essential in embryos but not in adults. In addition, the skeletal growth defect induced by *ACC1* knockout targeted to the adipose tissue revealed a systemic role in coordinating bone development.

The *Drosophila* model is a suitable system for investigating tissues-specific functions, given the availability of inducible RNA-interfering (RNAi) lines that can target the large majority of fly genes [28] and of genetic drivers to induce these RNAi in various tissues. The *Drosophila* genome contains a single *ACC* gene and three distinct *FAS* homologues (hereafter called *FAS^CG3523^*, *FAS^CG3524^*, *FAS^CG17374^*) [29]. In larvae, expression of the *FAS^CG3523^* gene has been detected in all tissues analyzed to date, whereas the *FAS^CG3524^* and *FAS^CG17374^* genes are essentially expressed in the carcass that include cuticle, epidermis, muscle and oenocytes [30]. For synthesis of VLCFA, the *Drosophila* genome encodes 20 Elongases, at least 2 TER homologues, and 2 potential HADCs, but only a single KAR gene product [29].

In order to assess the lipogenic function in *Drosophila*, we focused on the single *ACC* gene. As was the case with *ACC1* knockout mice, *ACC* mutant flies died as embryos. Moreover, similar to the conditional *ACC1* knockout targeted to the mouse liver or adipose tissue, FB disruption of the *Drosophila* ACC provokes metabolic perturbations but does not affect viability. In contrast, ACC disruption in the oenocytes induced a lethal phenotype similar to the one observed following genetic ablation of the oenocytes. We further provide evidence that this phenotype is due to failure of the synthesis of a putative VLCFA and results from a functional defect of lipid transfer within the spiracles. In summary, our findings show that, in the *Drosophila* larva, a putative VLCFA synthesized in the oenocytes induces a remote control necessary to maintain the watertightness of the respiratory system.

## Results

### ACC is a conserved lipogenic enzyme in *Drosophila*

Blast search using the mammalian *ACC1* or *ACC2* coding sequences as bait identified only a single homologous gene in the *Drosophila* genome (*CG11198*). The catalytic activity of ACC requires the activation of a carboxyl-donor group at the N-terminal domain and the transfer of this carboxyl group to the acetyl-CoA substrate at the C-terminal domain [17]. Amino acid alignment of the *Drosophila* and mammalian polypeptides indicates that these catalytic domains and their structural organization are tightly conserved (Figure S1). An available P-element (*PBac[5HPw^+^]CG11198^B131^*, hereafter referred to as *ACC^B131^*) inserted in the *ACC* gene likely results in a non-functional enzyme, since it interrupts the coding frame between the two main catalytic domains of ACC (Figure S2).

**Figure 1:**
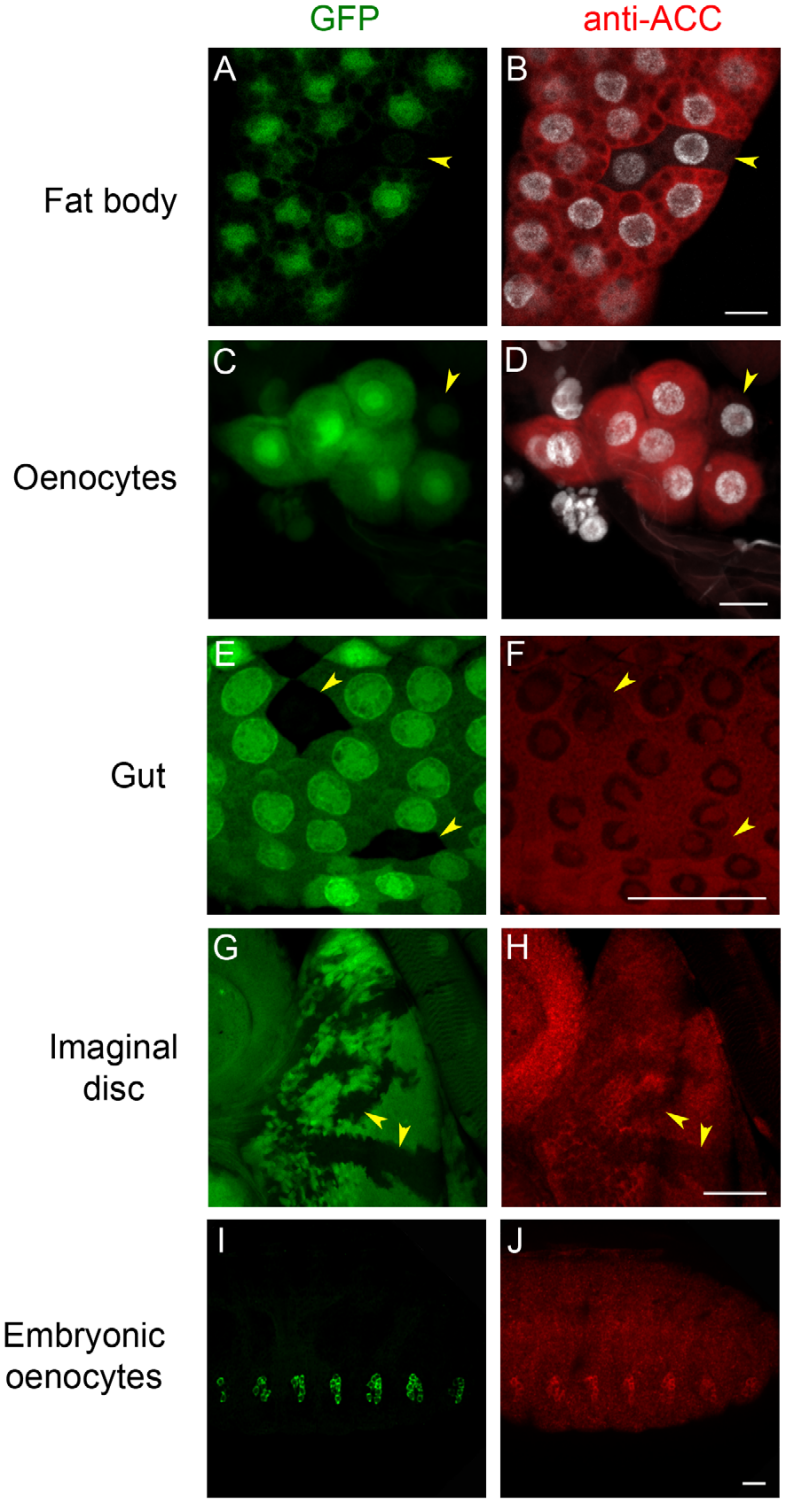
Immunodetection of ACC. Direct GFP fluorescence (A,C,E,G), and staining to ACC (B,D,F,H,J) and nuclei (B,D). ACC expression was very high in the FB (B) and in the oenocytes (D) of L3 larvae, and thus detection was performed with very low laser intensity. The ACC signal was lower in the gut (F) and in the imaginal discs (H) of L3 larvae, and thus detection was performed with high laser intensity. Specificity of the ACC signal was monitored by generating homozygote *FRT*-*ACC^B131^* clones (identified by the lack of GFP staining; arrowheads in A,C,E,G), which do not express the ACC protein in the corresponding pictures (arrowheads in B,D,F,H respectively). In the embryo, the strongest ACC staining was observed in the oenocytes (J) co-labeled to GFP driven by *BO-Gal4* (I). Scale bars: 20 µm.

To analyze ACC expression in *Drosophila* tissues, a rabbit antiserum was produced using peptides that correspond to coding sequences downstream of the *ACC^B131^* insertion site. The specificity of the immuno-labeling was analyzed on mosaic tissues in larvae that contain homozygous *ACC^B131^* mitotic clones. Consistent with the ubiquitous expression of the *ACC* mRNA [30], a cytoplasmic fluorescent signal was observed in most tissues (Figure 1 and data not shown). In addition, nucleolar labeling was visible, which is likely unspecific since it did not decrease in homozygous *ACC^B131^* clones (Figure 1). The strongest staining was observed in the cytoplasm of the FB cells and of the oenocytes (Figure 1A–1D), which is consistent with the storage and hepatic-like functions of these organs. A lower staining was detected in most larval tissues, as shown for the gut and the imaginal discs (Figure 1E–1H). In embryos, co-localization of an oenocyte GFP-reporter with the ACC signal indicates that the highest ACC levels are detected in the oenocytes (Figure 1I–1J). These findings emphasize the role of the oenocytes and of the FB in regulating FA homeostasis and prompted us to further investigate the function of ACC in these tissues.

Homozygous *ACC^B131^* mutants died at late embryogenesis (Table 1, line 1); the embryo failed to hatch and did not exhibit any apparent morphological defects (data not shown), indicating that *ACC* is an essential gene. Likewise, ubiquitous expression of an RNAi to *ACC* using the inducible *UAS/Gal4* system led to late embryonic lethality (Table 1, line 2). This system was very efficient at suppressing *ACC* expression, since a severe drop in ACC protein levels was observed in flip-out clones that expressed RNAi to *ACC* (Figure S3). To further assess the function of ACC in relation to FA metabolism, the *UAS-RNAi* line to *ACC* (*ACC-RNAi*) was induced with the FB-specific *Cg*-*Gal4* driver that is active throughout the larval and pupal stages (*Cg*-*Gal4* driving *UAS-ACC-RNAi*; hereafter called *Cg>ACC-RNAi*). No visible effect was observed on the development of larvae (Table 1, line 3).

**Table 1:** Genetic analysis of *ACC* by tissue-targeted knockdown.

Line	Genotype	Specificity	Phenotype
**1**	*ACC^B131^*	mutant	† embryo
**2**	*da-Gal4>ACC-Ri*	ubiquitous	† embryo
**3**	*Cg-Gal4>ACC-Ri*	FB	viable
**4**	*BO-Gal4>UAS-grim*	oenocytes	† @4–5d; L2/L3
**5**	*BO-Gal4>ACC-Ri*	oenocytes	† @4–5d; L2/L3
**6**	*BO-Gal4>ACC-Ri; svp-Gal80*	oenocytes	viable
**7**	*ACC^B131^; da-Gal4>UAS-ACC*	mutant	viable
**8**	*ACC^B131^; da-Gal4>UAS-ACC; svp-Gal80*	mutant	† @4–5d; L2/L3
**9**	*BO-Gal4>FAS^CG3523^-Ri*	oenocytes	viable
**10**	*BO-Gal4>FAS^CG3524^-Ri*	oenocytes	viable
**11**	*BO-Gal4>FAS^CG17374^-Ri*	oenocytes	† @4–5d; L2/L3
**12**	*BO-Gal4>KAR-Ri*	oenocytes	½† @4–5d; L2/L3
**13**	*BO-Gal4>CG6660-Ri*	oenocytes	† @4–5d; L2/L3

Column 1 (Line) lists the tests referred to in the text. Column 2 (Genotype) summarizes the mutant (*ACC^B131^*) or transgenic combinations used. Column 3 (Specificity) indicates the organ targeted by the *Gal4*-driver, or the use of the *ACC^B131^* insertion mutant. Column 4 (Phenotype) summarizes the resulting viability (viable), lethality (†) or semi-lethality (½†). The developmental stage at which death occurs is indicated; L2/L3 refers to animals at the second/third larval stage transition. @4–5d indicates the day of lethality after egg deposit.

**Figure 2:**
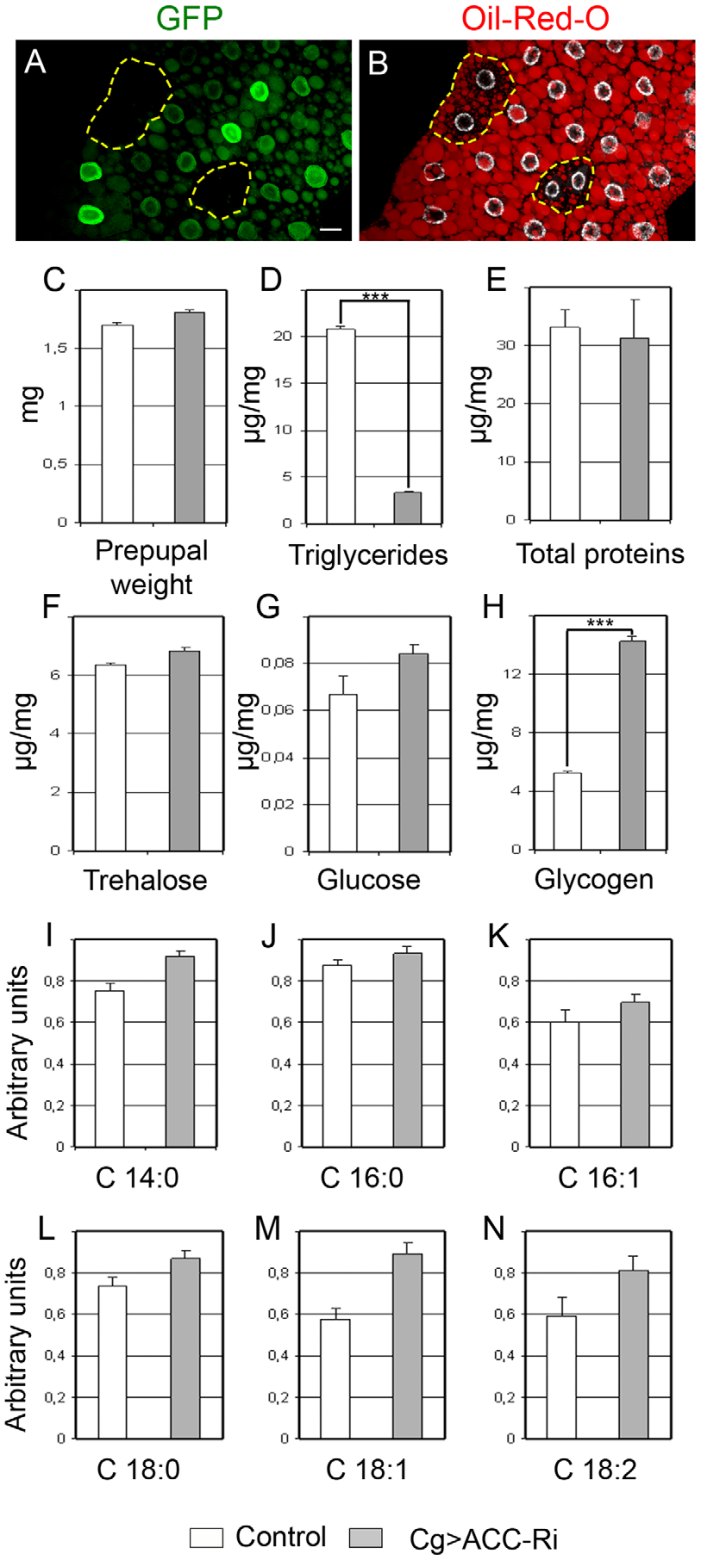
Metabolic defects due to ACC disruption. (A, B) LD contents labeled by Nile red staining (B) in the FB of well fed animals. Note the drop of LD accumulation (B) in homozygote *FRT-ACC^B131^* mutant clones marked by the absence of GFP (A); the dotted yellow line surrounds the GFP-negative clone (Scale bar: 20 µm). (C) Mean weight (mg) of 0–4 h prepupal female. (D–H) Concentration of TGs (D), proteins (E), trehalose (F), glucose (G) and glycogen (H) levels in prepupae. The values represent the concentration of each metabolite in µg per mg of 0–4 h prepupae. (I–N) Relative concentration (arbitrary units) of tetradecanoic (I), palmitic (J) palmitoleic (K), stearic (L), oleic (M) and linoleic (N) acid in 100 mg of 0–4 h prepupae. Color symbols (C–N): control (white bar) or expressing an *ACC-RNAi* in the FB (grey bar). T test: *: P<0.05; **: P<0.01; ***: P<0.001.

To evaluate the lipogenic role of ACC, we analyzed the lipid content of *ACC^B131^* somatic mutant clones in the FB. During the third larval stage (L3), the FB accumulates large amounts of LDs that dramatically dropped in *ACC^B131^* homozygous mutant clones (Figure 2A–2B), indicating that lipid stores depend, at least in part, on *de novo* synthesis inside the FB. The metabolic consequences of ACC disruption within the entire FB was then analyzed in 0–4 h prepupae; this is a convenient phase to tightly stage the animals after the feeding period. Surprisingly, *Cg>ACC-RNAi* pupae were not delayed in development (data not shown) and maintained a normal body weight (Figure 2C), though the total TG concentration dramatically dropped (Figure 2D). In these animals, protein concentration remained unchanged (Figure 2E). Further measurements revealed that neither glucose nor trehalose levels were severely affected (Figure 2F–2G), but that glycogen content increased dramatically in *Cg*>*ACC-RNAi* prepupae (Figure 2H). Most probably, the *Cg*>*ACC-RNAi* larvae compensate for the reduced TG storage through a metabolic switch to glycogen storage. Moreover, mass spectrometry analysis did not identify a significant decrease in the non-esterified FAs (NEFAs) detected in the prepupal extracts (Figure 2I–2N), suggesting that the NEFAs are not synthesized in the FB. Taken together, these findings indicate that the FB is a lipogenic organ and definitely confirm that ACC is a conserved enzyme required for FA synthesis.

### Fatty acid metabolism in the oenocytes

We next investigated the role of ACC in the oenocytes, which together with the FB exhibit the highest ACC expression levels (Figure 1B, 1D). Directing the *ACC-RNAi* with the oenocyte-specific *BO-Gal4* driver (*BO>ACC-RNAi*) induced a lethal effect at the L2/L3 larval transition (Table 1, line 5), similar to the phenotype reported after oenocyte ablation done by directing the pro-apoptotic gene *reaper* (*rpr*) [14]. Induction of *grim*, another pro-apoptotic gene, using the same *BO-Gal4* driver (*BO>grim*) also had a lethal effect at the L2/L3 larval stage transition (Table 1, line 4). Given that *BO-Gal4* is also active in a subset of neurons, Gutierrez and colleagues produced a transgenic line that expresses the Gal4 inhibitor, Gal80, driven by one of the enhancers of the *seven-up* promoter (*svp-Gal80*) that is characterized to be active only in the oenocytes [14]. Combining the *svp-Gal80* transgene with *BO-Gal4* retains *ACC-RNAi* expression in the neurons but not in the oenocytes. This results in a complete reversal of the lethal effect at the L2/L3 transition (Table 1, line 6), indicating that ACC plays an essential role in the oenocytes.

Considering that an RNAi generally represses but does not fully eliminate the mRNA target, we exploited the *ACC^B131^* mutation. The embryonic lethality of homozygous *ACC^B131^* animals can be rescued by ubiquitous expression of a transgenic *UAS-ACC* (Table 1, line 7), which definitely confirms that *ACC* is an essential embryonic gene. To formally demonstrate that the lethality of *BO*>*ACC-RNAi* animals was due to ACC disruption, we used the *svp-Gal80* transgene to impede the ubiquitous rescue of *ACC^B131^* homozygous mutant in the oenocytes. In this way, we generated tissue-specific mutants, hereafter referred to as *ACC-oenocyte-Targeted-Deficiency* (*ACC^oeTD^*). The *ACC^oeTD^* mutants exhibited a lethal phenotype at the L2/L3 larval stage transition (Table 1, line 8), which confirms that the *BO>ACC-RNAi* phenotype is due solely to the lack of *ACC* and further validates the use of *RNAi* lines to perform genetic tests.

Malonyl-coA is required for synthesis of both LCFAs and VLCFAs; a defect in LCFA or VLCFA synthesis may therefore be responsible for the lethal effect. LCFA synthesis is catalyzed by FAS that is encoded by three distinct genes in *Drosophila* (*FAS^CG3523^*, *FAS^CG3524^*, *FAS^CG17374^*), whereas VLCFA synthesis is catalyzed by a multi-protein elongase complex, of which only the KAR subunit is encoded by a single gene (*CG1444*). Therefore, we induced an RNAi to each of the three *FAS* genes and to *KAR* in the oenocytes with the *BO-Gal4* driver. In this setting, RNAi expression to *FAS^CG3523^* or *FAS^CG3524^* was fully viable (Table 1, line 9,10), whereas RNAi expression to *FAS^CG17374^* induced lethality at the L2/L3 transition (Table 1, line 11). *BO>KAR-RNAi* larvae also exhibit lethality at the L2/L3 transition, although a lot of escapers were observed (semi-lethal phenotype; Table 1, line 12) possibly because of an incomplete RNAi effect. Considering that the elongase subunits normally exhibit substrate and final product specificities, we induced the RNAi to each of the twenty elongases (data not shown) identified in the *Drosophila* genome using the *BO-Gal4* driver. In this genetic setting, only the RNAi to *CG6660* induced a lethal phenotype at the L2/L3 transition (Table 1, line 13). The phenotypes induced by the *ACC*-, *KAR*- and *CG6660-RNAis* shared strong similarities, indicating that the lethal effect depends on the failure of VLCFA synthesis.

### Oenocyte ACC regulates larval development and feeding behavior

**Figure 3:**
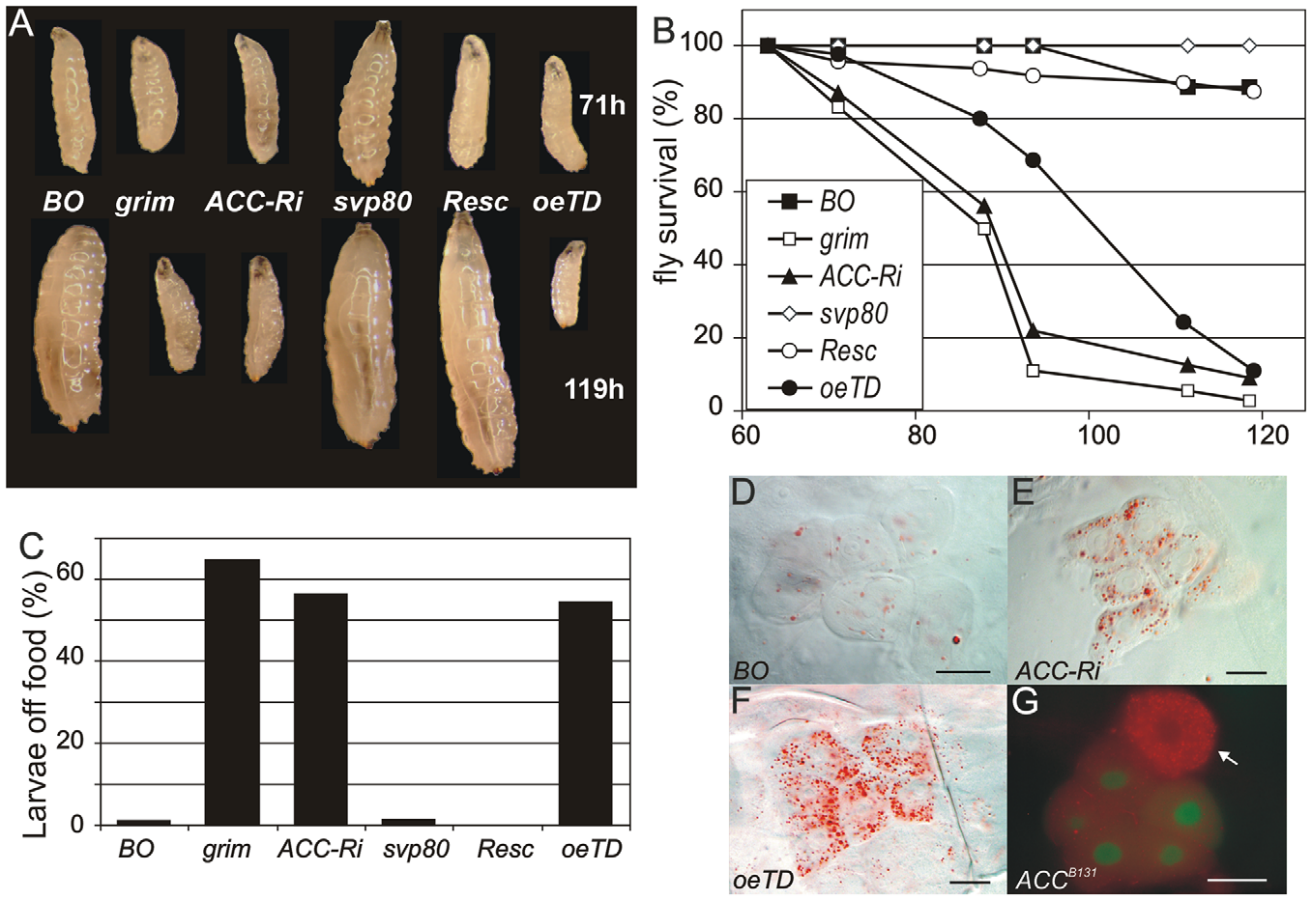
ACC disruption in the oenocytes. (A) Growth phenotype at the L2/L3 transition (larvae at the top of the panel) and 2 days later (larvae at the bottom of the panel) corresponding to 71 h- and 119 h-old larvae, respectively. Symbols used: (*BO*) *BO-Gal4*/+ control; (*grim*) *BO>grim*; (*ACC-Ri*) *BO>ACC-RNAi*; (*svp80*) *svp-Gal80;BO>ACC-RNAi*; (*Resc*) *ACC^B131^;da-Gal4>UAS-ACC, ACC* mutant rescued by ubiquitous *ACC-cDNA* expression; (*oeTD*) *ACC^oeTD^*, *ACC* mutant targeted to the oenocytes. The differences in size observed at 71 h are not representative of all individuals; 71 h is a median value after a 5 h collection of newly-hatched larvae. (B) Survival of animals in hours after egg-laying. Symbols used as in A: (black square) *BO-Gal4*/+ control; (empty square) *BO>grim*; (black triangle) *BO>ACC-RNAi*; (empty diamond) *svp-Gal80;BO>ACC-RNAi*; (empty circle) *ACC^B131^;da-Gal4>UAS-ACC*; (black circle) *ACC^oeTD^*. (C) Feeding behavior of late L2 larvae. The percentage indicates the proportion of larvae that stray away from the food 1 h after being placed in a small piece of food in the middle of an agar plate. Symbols used as in A. (D–G) Oenocytes stained with Oil-Red-O. The oenocytes of *BO>ACC-RNAi*; (E) and *ACC^oeTD^* (F) late L2 larvae accumulate high levels of LDs compared to control oenocytes (D). (G) An *ACC^B131^* mutant oenocyte labeled by the absence of GFP (arrow) accumulate high levels of LDs compared to the neighboring control oenocytes (GFP labeled). Scale bars: 20 µm.

We then compared the phenotypes of *BO>grim BO>ACC-RNAi* and *ACC^oeTD^* animals, which all die at roughly the L2/L3 transition (Figure 3A). Larvae of either genotype grew normally until the end of the second stage but were unable to grow further. They survived a few more days but eventually contracted in size (Figure 3A) before dying between 4 to 5 days after egg deposit (Figure 3B). As previously reported for oenocyte ablation [14], the *BO>grim* and *BO>ACC-RNAi* larvae often exhibited duplicated mouth hooks of L2 and L3 identity, although some larvae completed the L2/L3 transition. *ACC^oeTD^* animals also die at the L2/L3 transition but were able to complete the molting process, whereas *ACC^B131^* mutants rescued by *UAS-ACC* ubiquitous expression developed to adulthood (Figure 3A, 3B and data not shown). These findings confirm that oenocyte ACC is required for the proper development past the L2/L3 transition.

As reported for oenocyte-ablated larvae [14], we also observed a peculiar feeding behavior in *BO*>*ACC-RNAi* and *ACC^oeTD^* larvae. At the end of the L2 stage, control larvae continued foraging inside the food, while more than 50 percent of *BO*>*ACC-RNAi*, *ACC^oeTD^* and *BO*>*grim* larvae strayed away from the food (Figure 3C). Intriguingly, all of these deficient larvae exhibited increased motility, although the larvae ultimately stopped moving several hours before death (data not shown). Taken together these findings indicate that the phenotype induced by oenocyte ablation can be recapitulated by ACC-disruption and is likely due to the subsequent lack of VLCFA synthesis in the oenocytes.

### ACC disruption-induced lethality is independent of LD uptake in oenocytes

To investigate the cell-specific roles of ACC, oenocytes of *BO*>*ACC-RNAi* and *ACC^oeTD^* larvae were observed at the late L2 larval stage. The oenocytes were clearly visible in both genetic settings but, surprisingly, accumulated high amounts of LDs (Figure 3D–3F) similar to the phenotype previously reported after overnight fasting [14]. This phenotype depends on a cell-autonomous response since a dramatic increase of LD content was observed both in homozygous *ACC^B131^* somatic clones (Figure 3G) and in flip-out clones expressing *ACC-RNAi* (Figure S4). The accumulation of LDs in ACC-deficient oenocytes likely results from lipid uptake rather than *de novo* synthesis. Expression of the lipophorin receptors *LpR1* (*CG31094*) and *LpR2* (and *CG31092*) has previously been reported in oenocytes [14]. Therefore, we generated deficient mutants for either of them (Figure S5) and, in agreement with another study [31], both were homozygous viable (unpublished results). Analysis of oenocytes after overnight starvation revealed that LD uptake proceeded normally in *LpR1*-deficient mutants, but failed both in *LpR2*-deficient mutants (data not shown) and in flip-out oenocytes-expressing *LpR2-RNAi* (Figure S4). Next, RNAis to several potential lipophorin receptors (*LpR1, LpR2, CG8909, CG33087*) were co-induced with *ACC-RNAi* in flip-out clones. In this way, clones that co-expressed RNAi to *LpR2* and *ACC* failed to accumulate LDs (Figure S4), whereas clones that coexpressed RNAi to *ACC* and to any of the other receptors did accumulate LDs (data not shown). Finally, homozygous *ACC^B131^* clones were generated in an *LpR2* mutant background. When observed in the oenocytes, these *ACC^B131^* clones failed to accumulate LDs (Figure S4). Taken together, these findings indicate that LpR2 is required for cell-autonomous LD uptake into the oenocytes induced by either starvation or ACC deficiency. Consequently, the lethality of *BO*>*grim* and *BO*>*ACC-RNAi* larvae is likely independent of the LD uptake, since the oenocytes of the former are ablated, while in contrast, those of the latter dramatically increase their LD content.

### ACC is required for integrity of the respiratory system

**Figure 4:**
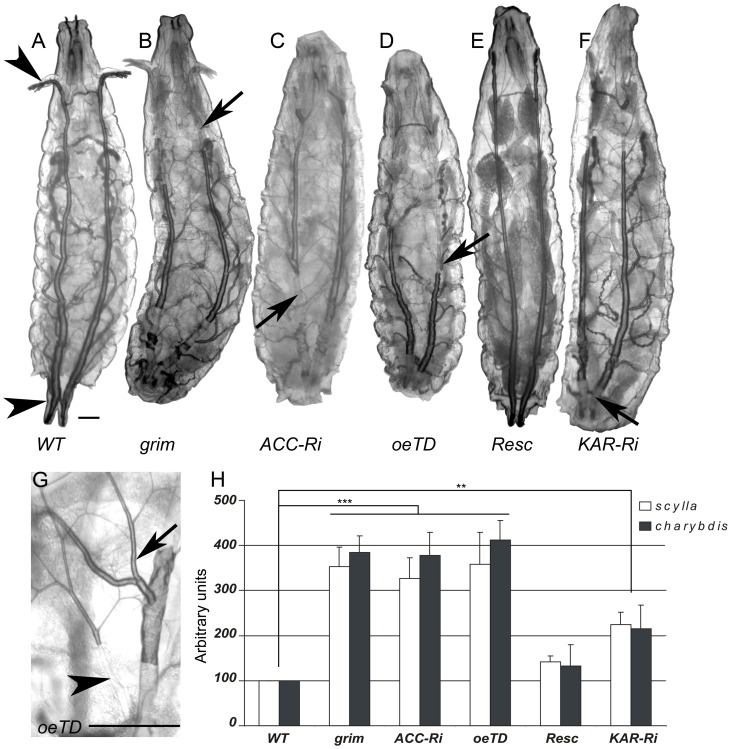
Oenocytes ACC signals to the tracheal system. (A) Dorsal view of a wild-type larva showing the two dorsal main trunks of the tracheal system, which extend from the anterior to the posterior spiracles (arrowheads). Air-filling phenotypes in the tracheal systems of *BO>grim* (B), *BO>ACC-RNAi* (C), *ACC^oeTD^* mutant (D), *ACC^B131^* mutant rescued by ubiquitous *UAS-ACC* (E) and *BO>KAR-RNAi* (F) animals. The portions of the dorsal main trunks filled with an aqueous solution are difficult to distinguish (arrows). The larvae have been selected early after the L2/L3 molting transition (prior to death in B,C,D,F). Larvae oriented anterior to the top. (G) Higher magnification of tracheal branches in early L3 *ACC^oeTD^* larvae; a branch that originates from the air-filled part of the main trunk is entirely filled with air (arrow) whereas a branch that originates from the liquid-filled part of the main trunk is filled with liquid in its proximal end but not in the distal sub-branches (arrowhead). Scale bars: 100 µm. (H) Transcriptional expression of the *charybdis* and *scylla* hypoxic-responsive genes in control, cell-ablated, ACC or KAR deficient animals. Symbols used: (*WT*) control; (*grim*) *BO>grim*; (*ACC-Ri*) *BO>ACC-RNAi*; (*oeTD*) *ACC^oeTD^*; (*Resc*) *ACC^B131^;da-Gal4>UAS-ACC*; (*KAR-Ri*) *BO-Gal4>KAR-RNAi*. Genotypes behaved differently in their hypoxic response (ANOVA: genotype: F5,32 = 36.19 P<10^−3^; gene F1,32 = 1.24 P = 0.27; genotype×gene: F5,32 = 0.47 P = 0.795). Dunnet T test: *: P<0.05; **: P<0.01; ***: P<0.001.

The peculiar behavior of *BO*>*ACC-RNAi* and *ACC^oeTD^* larvae, characterized by food repulsion and increased motility followed by an inactive phase, bears a striking resemblance to the phenotype reported for oxygen-deprived larvae [32]. We therefore examined the trachea of larvae of various genotypes. The tracheal system of wild-type larvae is readily visible, because air-filled respiratory tubules are highlighted by thick, dark lines (Figure 4A). The tracheal network of *BO*>*grim*, *BO*>*ACC-RNAi*, and *ACC^oeTD^* animals appeared normally filled with air at the L1 stage (data not shown), while at the L2/L3 transition the larvae found outside of the food exhibited a severe default in air-filling of their tracheal trunks (Figure 4B–4D). The tracheal tubes were still distinguishable, suggesting that they were present but filled with an aqueous solution (Figure 4B–4D). The air-filling failure was observed in the spiracles and in the main trunks, but only occasionally extended into the lateral branches and the tracheoles (Figure 4G). Conversely, the *ACC^B131^* mutant rescued by ubiquitous expression of the *UAS-ACC* (Figure 4E), as well as the *BO*>*ACC-RNAi* animal expressing the *svp-Gal80* transgene (Figure S6), exhibited a normally air-filled tracheal network. This tracheal phenotype relies on VLCFA synthesis, since air-filling failures were also visible in L2/L3 larvae expressing the RNAi to *KAR*, *CG6660*, and *FAS^CG17374^* in their oenocytes (Figure 4F and Figure S6). A closer analysis revealed that a few of the mutant larvae remained alive inside the food, and these animals had only one or two defective spiracles (data not shown). In contrast, mutant larvae that strayed away from food exhibited either an air-filling defect in all four of their spiracles or major liquid-filling of at least one of the main trunks. We further eliminate the possibility that this phenotype directly depends on an abnormal molting process at the L2/L3 transition, since a few of the mid/late L2 larvae with the *BO*>*grim* and *BO*>*ACC-RNAi* genotypes exhibited air-filling defects of their tracheal system (Figure S6).

Examination of GFP-induced expression revealed that the *BO-Gal4* driver was also active in a few spiracle cells that face the tracheal lumen (data not shown). To exclude a potential role of these spiracle cells in the lethal phenotype, we made use of the recently published *promE-Gal4* driver reported to be oenocyte-specific [33]. Systematic observations of GFP-induced expression revealed that the *BO-Gal4* driver was active in embryonic but not in larval oenocytes, while in contrast, the *promE-Gal4* driver was active in larval but not in embryonic oenocytes (data not shown). Nonetheless, the *promE-Gal4* driver was never active in the spiracles (data not shown). As for *BO>grim* animals, directing the proapoptotic gene *grim* with the *promE-Gal4* driver induced a similar lethal phenotype associated with tracheal defects at the L2/L3 transition (Figure S6). Moreover, animals directing RNAi to *ACC, FAS^CG17374^* or the *CG6660* elongase with the *promE-Gal4* driver were able to successfully complete the L2/L3 molting process. A few with a tracheal liquid-filling phenotype died at the early L3 stage (Figure S6), but most exhibited tracheal defects at the mid L3 stage, dying at either the late L3 stage or in their pupal case (data not shown). The higher phenotypic penetrance obtained with the *BO-Gal4* driver might be a consequence of the earlier Gal4 activity, since *BO-Gal4* is active during embryogenesis while *promE-Gal4* become active at the L1 stage. Nevertheless, the similarity of the phenotypes induced by either driver definitely confirms that a specific VLCFA synthesized in the oenocytes is necessary to maintain the integrity of the tracheal system.

### Metabolic defects and oenocyte dysfunction

The observed tracheal phenotype strongly supports the notion that the larvae died of anoxia as a consequence of respiratory failure. To investigate this issue, we used quantitative RT-PCR to analyze the expression of the *Drosophila* REDD1 homologues *charybdis* and *scylla* known to be transcriptionally induced by hypoxia [34]. In agreement with the wild-type air-filling phenotype observed in L1 larvae, *charybdis* and *scylla* expression was not modified in mid L1 larvae with the *BO*>*ACC-RNAi* and *BO*>*grim* genotypes (Figure S7). In contrast, *charybdis* and *scylla* were dramatically induced in larvae at the L2/L3 transition (Figure 4H). Similarly, high expression levels of both genes were observed in *ACC^oeTD^* larvae but not in *ACC^B131^* mutant larvae rescued by ubiquitous expression of *UAS-ACC* (Figure 4G). Consistent with the semi-lethal phenotype, *charybdis* and *scylla* were also induced in *BO>KAR-RNAi* larvae at the L2/L3 transition, though at intermediate levels (Figure 4H). Taken together, these findings indicate that disrupting the synthesis of a specific VLCFA in the oenocytes provokes an air-filling failure of the tracheal system, thereby inducing a lethal anoxic phenotype.

As previously reported, oenocyte-ablated larvae are unable to use their TG stores during fasting [14]. To determine whether this was also the case for *BO*>*ACC-RNAi* animals, mid/late L2 larvae (40–45 h after hatching) were starved for 14 h. These larvae, like the *BO*>*grim* larvae, were unable to use their TG stores (Figure S7). However, we observed that late L1 larvae (19–24 h after hatching) of either genotype were able to use part of their TG stores (Figure S7). Moreover, late L1 *BO*>*ACC-RNAi* and *BO*>*grim* larvae expressed *charybdis* and *scylla* at normoxic levels (Figure S7) and did not exhibit any tracheal defect (data not shown). In contrast, these larvae at the mid/late L2 stage expressed *charybdis* and *scylla* at hypoxic levels; a few of them exhibited minor tracheal defects before fasting and this phenotype increased dramatically over the 14 h of starvation (data not shown). Since oxidation of FAs is an oxygen-dependent process, it is questionable whether the lack of TG consumption in *BO*>*ACC-RNAi* and *BO*>*grim* larvae solely reflects the function of oenocytes in TG consumption or whether it also results from a subsequent effect of oxygen deprivation.

### Oenocytes control spiracle activity

**Figure 5:**
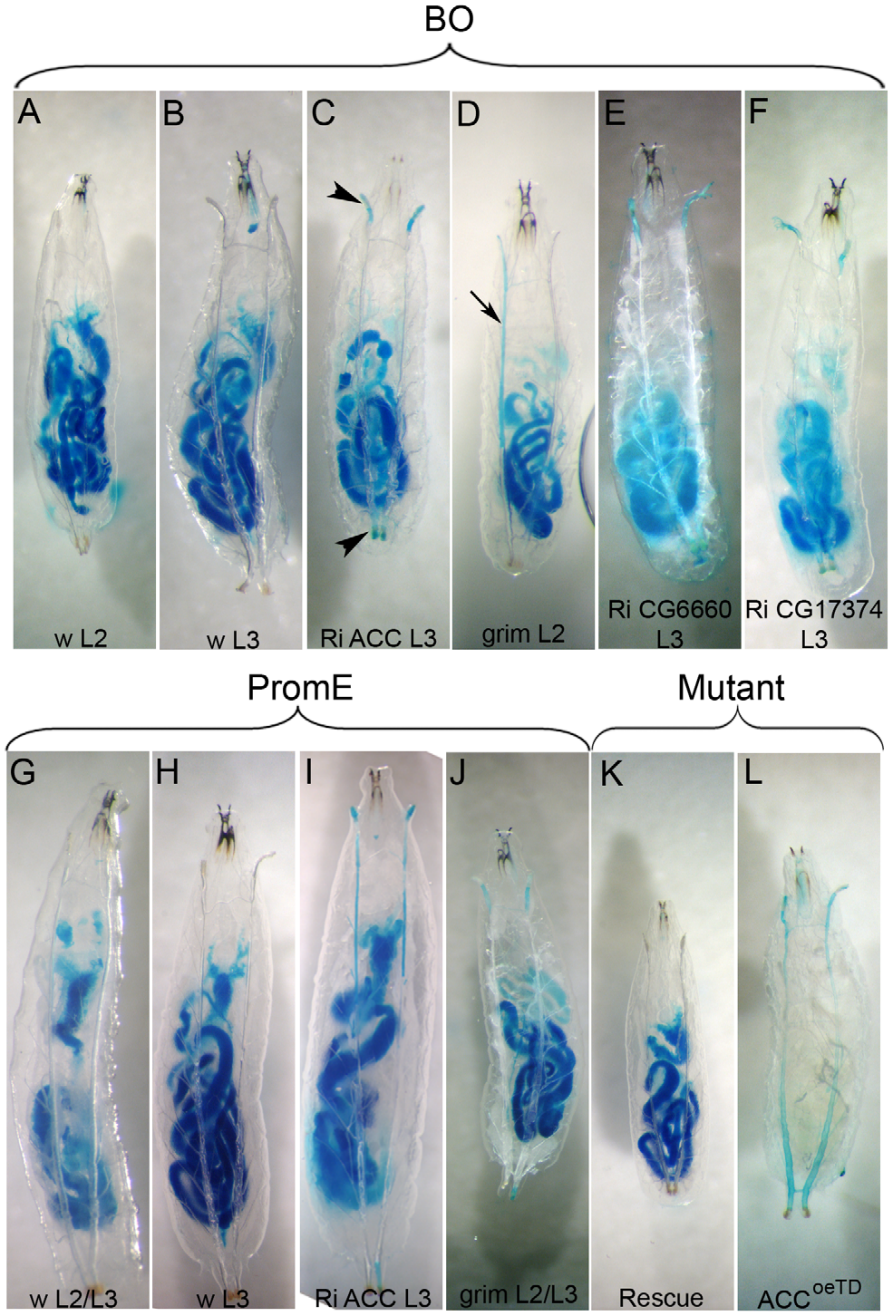
Water-tightness defect of the spiracles. (A–L) Tests for liquid entry into the tracheal system after transfer of larvae onto a semi-liquid media stained with Brilliant Blue FCF. Control larvae were maintained for 36 h inside the tinted feeding medium and analyzed at various times (A,B,G,H,K) Mutant larvae without any visible tracheal defect were transferred onto the tinted feeding medium and analyzed as they strayed away (C,D,E,F,I,J,L). Larvae of the following genotypes and stages: (A) *BO* control late L2, (B) *BO* control early L3, (C) *BO>ACC-RNAi* early L3, (D) *BO>grim* late L2, (E) *BO>CG6660-RNAi* early L3, (F) *BO>FAS^CG17374^-RNAi* early L3, (G) *promE* control at the L2/L3 transition, (H) *promE* control early L3, (C) *promE>ACC-RNAi* early L3, (D) *promE>grim* at the L2/L3 transition, (K) *ACC^B131^* mutant rescue and (L) *ACC^oeTD^*. Note that the staining of the gut due to the tinted food is not visible in the hemolymph, which confirms that Brilliant Blue FCF does not easily cross the epithelia. Entry of tinted liquid into the anterior and posterior spiracles (arrowheads in C) and extension into the main trunks (arrow in D) are indicated. Larvae oriented anterior to the top.

The tracheal defect observed in this study primarily affected the spiracles and the tracheal trunks; it has little effect on the tracheal branches and the tracheoles. To determine whether the liquid that accumulated in the tracheal system originated from the feeding medium, the food was stained with the brilliant blue FCF dye. Control larvae at the L2 or L3 stages maintained in the tinted food over 36 h exhibited no staining of their tracheal system or of their hemolymph (Figure 5A–5B, 5H), even when observed during the molting process (Figure 5G). Mutant larvae at the L2 or early L3 stages were transferred into the tinted food, prior the onset of the spiracle defect, and analyzed as they strayed away from the media. Larvae directing the RNAi to *ACC, FAS^CG17374^*, the *CG6660* elongase, or the *grim* transgene with the *BO-Gal4* driver exhibited blue staining of their spiracles that sometimes extended into the main tracheal trunks (Figure 5C–5F). Liquid filling was sometimes visible in late L2 or early L3 larvae or at the L2/L3 transition. Directing the *grim* transgene or *ACC-RNAi* with the *promE-Gal4* driver induced liquid filling of the spiracles and of the tracheal trunks, mostly at early L3 stage although it sometimes occurred at the L2/L3 transition (Figure 5I–5J). In this experimental setting, the spiracles and the tracheal trunks of *ACC^oeTD^* animals were also filled with blue-tinted liquid, a phenotype that could not be observed in *ACC^B131^* mutants rescued by ubiquitous expression of the *UAS-ACC* (Figure 5K–5L). Nonetheless, we never observed staining of the hemolymph, indicating that the tinted liquid could not cross the epithelia. Taken together, these findings indicate that the liquid comes from the semi-liquid feeding media and enters through dysfunctional spiracles.

### Oenocyte ACC controls lipid transport within the spiracles

**Figure 6:**
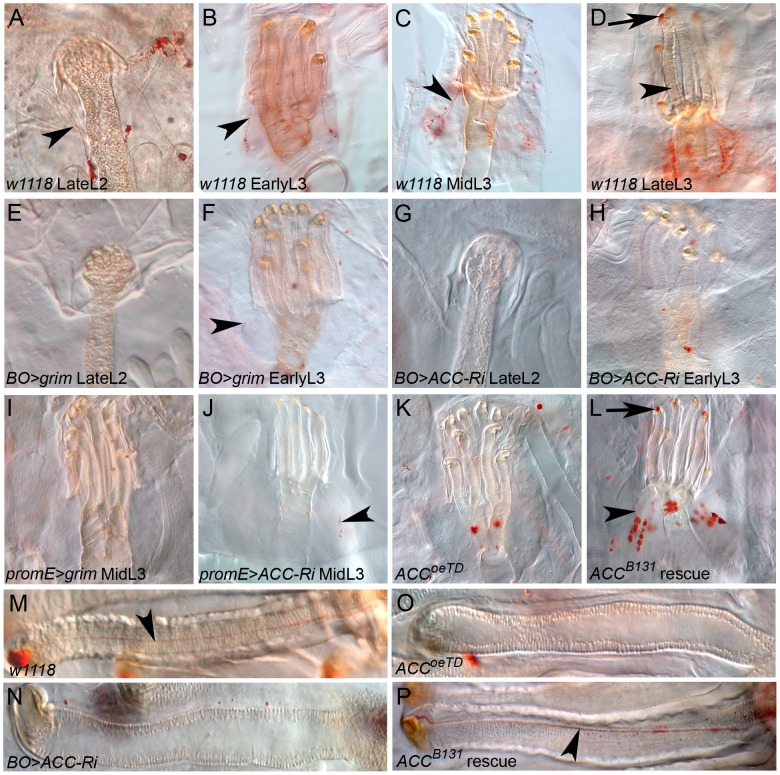
Lipid transfer within the anterior spiracles. (A–P) Oil-Red-O staining of anterior spiracles. (A–D) Control larvae at the late L2 (A), early L3 (B), mid L3 (C), late L3 (D) stages contain LD clusters in their spiracular glands that form lipid-containing ducts (arrowheads); note the accumulation of lipids at the spiracular opening of late L3 larvae (arrow in D). (F–L) Spiracles at the late L2 stage (E,G) and after the L2/L3 molt (F,H,I,J,K,L) of the following larvae: (E–F) *BO>grim*, (G–H) *BO>ACC-RNAi*, (I) *promE>grim*, (J) *promE>ACC-RNAi*, (K) *ACC^oeTD^* and (L) *ACC^B131^* mutant rescue. (M–P) Presence (M,P) or absence (N,O) of lipid-containing ducts in L3 spiracular terminal branch of the following larvae: (M) control, (N) *BO>ACC-RNAi*, (O) *ACC^oeTD^*; (P) *ACC^B131^* mutant rescue.

**Figure 7:**
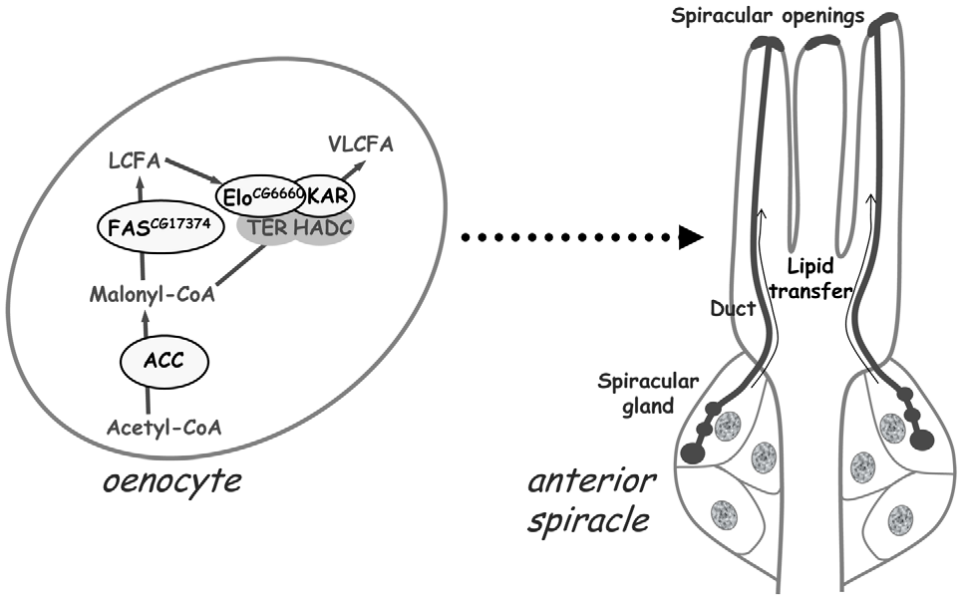
A VLCFA–dependent remote signal from the oenocytes controls lipid transfer within the spiracles. The default of VLCFA synthesis within the oenocytes provokes the failure to transfer lipids through the spiracular ducts from the spiracular gland to the spiracular opening. The gene products identified to be involved in this metabolic pathway within the oenocytes are indicated (oval forms surrounded in black). The signal running from the oenocytes to the spiracles is yet unidentified.

We carefully analyzed the spiracles to further investigate the tracheal defect induced by loss-of ACC in the oenocytes. Previous studies have determined that lipids are produced in the spiracular glands and transferred through ducts to the cleft of the spiracles [35], [36]. We therefore analyzed lipids in the spiracles by Oil-Red-O staining from the late L2 to late L3 stages. Variable LD levels were observed in the spiracular glands that were easier to distinguish at the anterior spiracles than at the posterior spiracles (data not shown). In late L2 larvae, these lipids form strings that run through fine ducts from the spiracular gland to the tip of the spiracle (Figure 6A). In L3 larvae, an anterior spiracle contains eight protruding branches [16]. For each of them, a cluster of LDs in the spiracular gland produces a duct that runs along the branch to reach the spiracular opening (Figure 6B–6D, 6M). The amounts of lipids, both in the spiracular glands and at the spiracular opening, increase during the third larval stage to reach a maximum in late L3 larvae (Figure 6B–6D). Disrupting ACC in the oenocytes provoked a dramatic phenotype in the spiracles. In *BO*>*ACC-RNAi* and *BO*>*grim* L2 larvae that still exhibited fully air-filled trachea, the LDs in the spiracular gland and the lipid-containing ducts were barely visible (Figure 6E, 6G), indicating that a functional defect of the spiracles occurs before the tracheal air-filling phenotype. Consistently, the *BO*>*ACC-RNAi* and *BO*>*grim* larvae that successfully completed the L2/L3 molting transition shared a loss of LD clusters and lipid-containing ducts in their spiracles (Figure 6F, 6H, 6N). A similar phenotype was observed in early L3 larvae of *promE*>*grim* and *promE*>*ACC-RNAi* genoptypes (Figure 6I, 6J). However, few ducts eventually formed but these hardly reached the tip of the spiracular branches (arrowheads in Figure 5F, 5J). *ACC^oeTD^* larvae contained neither LD clusters nor lipid-containing ducts in their spiracles (Figure 6K, 6O), whereas *ACC^B131^* mutant larvae rescued by ubiquitous expression of *UAS-ACC* exhibited a control-like phenotype (Figure 6L, 6P). Therefore, we concluded that a specific VLCFA must be synthesized in the oenocytes in order to remotely control the watertightness of the spiracles (Figure 7).

## Discussion

We have identified a *Drosophila ACC* that is likely the genuine and unique fruitfly member. First, blast search using the mammalian *ACC1* or *ACC2* as bait identified a single gene in the *Drosophila* genome. Second, the degree of identity and similarity between the *Drosophila* and mammalian ACC gene products is very high, as is the conservation of the various functional domains. Third, disruption of this single gene in the FB induces a dramatic drop in TG storage, indicating that *CG11198* is necessary for FA synthesis. Fourth, mutation of this unique *Drosophila ACC* gene causes embryonic lethality, as in the *ACC1* knockout mice that die at early embryogenesis [21]. Furthermore, we observed that disruption of oenocyte ACC phenocopies oenocyte ablation indicating that ACC fulfills an essential function of these cells. We also provide evidence that this phenotype depends on the synthesis of at least one unidentified VLCFA within the oenocytes. Importantly, we observed that the oenocytes control the watertightness of the trachea, and that this process is linked to the failure of lipid transfer to the apertures of air entry into the respiratory system. Based on these findings, we propose that the watertightness of the tracheal system depends on VLCFA synthesis within the oenocytes to control, through a yet unknown process, the formation of LD clusters in the spiracular gland and their transfer to the spiracular cleft (Figure 7).

Accumulation of LDs in the FB is assumed to be mediated by lipophorin receptor-dependent uptake from the hemolymph [37]. Nonetheless, our study in FB cells revealed that ACC is necessary for *de novo* synthesis of FAs and the subsequent TG storage, indicating that the FB is also a lipogenic organ. In mammals, conditional *ACC1* knockouts to the mouse liver [24] or adipose tissue are viable [25]. Interestingly, *ACC-RNAi* disruption in the FB did not affect viability, but did result in a severe drop in TG accumulation and a dramatic increase in glycogen levels. This phenotype directly depends on LCFA synthesis, since expression of an RNAi to the ubiquitous *FAS* gene *CG3523* in the FB produced comparable metabolic changes (TR and JM, unpublished results). The metabolism of TGs and glycogen plays a central role in homeostasis and in related diseases [38], [39]. Metabolic syndrome and type 2 diabetes are associated with increased levels of circulating NEFA, TGs, glucose, and with restricted postprandial storage of glycogen [40]. Accordingly, recent studies have revealed that increasing synthesis of glycogen in diabetic animal models is beneficial for reducing high concentrations of circulatory glucose [41], [42]. Most likely, the increased glycogen levels in *Drosophila* larvae that are unable to synthesize TGs in their FB represents a metabolic response aimed to counteract a potential diabetes-like threat. This extra glycogen potentially accumulates in the body wall muscles as it normally does in wild-type *Drosophila* larvae [13], suggesting a systemic control of the TG/glycogen storage balance. In mammals, accumulation of glycogen depends on the activity of two opposing enzymes: glycogen phosphorylase (GP) and glycogen synthase (GS), which catalyze glycogen breakdown and synthesis, respectively [39]. Insulin stimulation results in the repression of GSK3, thereby promoting synthesis of glycogen [43], as GS activity is inhibited by GSK3-induced phosphorylation [39]. The increased glycogen levels in ACC-deficient larvae likely results from a regulatory loop improving GS activity that may be mediated through insulin signaling. Alternatively, a recent study in mammals revealed that the Fibroblast-growth-factor 19 (FGF19) also induces GSK3 phosphorylation in an insulin-independent manner, thereby increasing GS activity [41]. Three FGF ligands (called Branchless, FGF8-like1 and FGF8-like2) have been identified in the *Drosophila* genome [44], [45]. Whether one of them also regulates glycogen metabolism, much like the mammalian FGF19, remains to be investigated.

Our findings emphasize the role of the oenocyte in FA metabolism. Earlier studies revealed that the oenocytes are closely associated with the FB cells and that both cell types undergo concurrent morphological changes [46]. These physiological relationships have recently been substantiated through the identification of a subset of gene products that act equally in either the FB or the oenocytes to control overall TG content [47]. Furthermore, as is the case in fast-induced fatty-liver, the oenocytes accumulate large amount of LDs following overnight starvation [14], a dynamic process that we also observed in ACC-deficient oenocytes. In mammals, the uptake of LDs in fasted liver is possibly controlled by low FA levels, since FASKOL mice that are fed a fat-free diet, accumulate more hepatic LDs than controls [27]. We observed that the uptake of LDs into the oenocytes, induced by either fasting or ACC deficiency, is mediated by the lipophorin receptor LpR2. Since both processes are expected to drop malonyl-CoA levels and FA synthesis, it is likely that this LpR2-mediated LD uptake is induced by a subsequent metabolic defect rather than by a direct effect of nutrients or ACC decrease. Furthermore, it has been shown that oenocyte-ablated larvae were unable to use their TG stores [14], suggesting that the oenocytes are the only tissues where TG remobilization takes place. However, we observed a striking correlation between TG consumption and hypoxia following oenocyte ablation, consistent with the fact that FA oxidation is an oxygen-dependent process [48]. These findings raise the question of whether the failure of TG consumption in oenocyte-ablated larvae is due to an autonomous function of the oenocytes or whether it results from a subsequent effect of hypoxia. In mammals, fasting induces remobilization of TGs from the adipose tissue to the lean tissues—namely the liver and the muscles [49]. Hence, in *Drosophila,* the idea that the muscles are unable to burn TG stores and that the oenocytes are the only cells able to do this might be reconsidered.

Here, we provide evidence that the essential function of the oenocytes does not depend on lipid remobilization. Instead, it relies directly on ACC activity to sustain the synthesis of a putative VLCFA that is required to preserve the watertightness of the spiracles (Figure 7). *De novo* synthesis of VLCFA is catalyzed by the elongase complex, whose elongase subunit is specific to the tissue, the substrate and the final product [20]. Using specific RNAi to each of the twenty *Drosophila* elongase subunits, we have found that only one of them could recapitulate the phenotype due to oenocyte disruption of ACC, arguing for a particular VLCFA in this essential function. Interestingly, a transcriptome analysis revealed that several genes encoding FA anabolic enzymes are upregulated in mosquito oenocytes, including FAS and two putative elongases [50]. Desat1, the *Drosophila* homologue of the Stearoyl CoA-desaturase-1—which catalyzes the formation of a double bound to generate the unsaturated palmitoleic and oleic acids—is one of the gene products reported to be highly expressed in oenocytes [51]. Strikingly, *desat1* mutation induces a lethal phenotype at the end of the L2 stage [52]. However, we have observed that larvae expressing a ubiquitous *desat1* RNAi die at the end of the L2 stage without any visible tracheal defect. In addition, larvae expressing this *desat1* RNAi in their oenocytes, survived to adulthood without any visible defective phenotype (data not shown), suggesting that the production of the putative VLCFA does not require Desat1. Nevertheless, identification of this VLCFA will be a substantial challenge that will require a combination of extensive *in vitro* and *in vivo* approaches.

For many decades, it has been proposed that lipid metabolism is highly active in the oenocytes of most insect species, to sustain the renewal of the cuticular lipids and their hydrocarbon derivatives at the molting transitions (references in [53]). In addition, molting defects and impairment of cuticle formation have been observed in *Caenorhabditis elegans* following knock down of the *pod-2* and *fasn-1* genes that encode ACC and FAS, respectively [54]. Nevertheless, in spite of a lethal effect that may happen at the L2/L3 transition, our findings do not formally demonstrate that VLCFA synthesis in the oenocyte is crucial for the larval molting processes. Actually, we have observed that disrupting VLCFA synthesis in the oenocytes induces an entry of liquid into the tracheal system at various developmental phases—from mid-L2 to L3 stages, depending on the genotype. Although default in removal of the tracheal cuticle was observed in dying larvae blocked at the L2/L3 transition, no obvious defect was visible when the watertightness failure arose during the L2 or L3 stages (data not shown). Thus, the spiracle defect is likely independent of the molting process failure.

Finally, we show that disruption of VLCFA synthesis in the oenocytes is associated with a lack of both LD clusters in the spiracular gland and lipid-filled ducts running to the spiracular opening. Formation of LDs in the spiracular gland and lipid transfer to the spiracular aperture was described several decades ago in various insect species [35]. The proposed explanation for lipid accumulation is that it functions to prevent liquid entry into the tracheal system of aquatic insects. In contrast, terrestrial insects must be resistant to water loss, a process that can occur through the cuticle and the spiracles [55]. Although the adult *Drosophila* is a terrestrial insect that needs a barrier to protect it from desiccation, the larva is a semi-aquatic animal [56]. Our findings provide the first genetic evidence that failure to transfer lipids to the air entry opening is linked to a loss of the hydrophobic properties of the larval spiracles. The accumulation of liquid in the tracheal system has also been reported following clearance failure of the tracheal lumen. Few gene products have been described for which mutations result in liquid accumulation in the trachea. These gene products comprise epithelial Na+ channels (ENaCs) [57], a J-domain transmembrane protein [58] and a putative transmembrane protein of unknown function [59]. Defective lung liquid clearance is responsible for several human diseases that manifest at different ages [60]. Interestingly, mice that are deficient in one of the ENaC family members (α-ENaC) die because of defective neonatal liquid clearance in the lungs [61]. To date, all the *Drosophila* mutants that produce the gas-filling defect induce an early phenotype visible in embryo and L1 larvae. The corresponding gene products are expressed in the tracheal cells and apparently work in a tissue-autonomous manner. In contrast, we never observed the oenocyte *ACC*-dependent phenotype earlier than the late L2 stage, suggesting that it originates from a distinct physiological event. Given that the *BO-Gal4* driver is active in embryonic but not in larval oenocytes, synthesis of this putative VLCFA is likely critical during early development, while the tracheal defects and lethality arise later. Therefore, this VLCFA-dependent signal could either send an early message to the embryonic trachea, or be necessary for proper oenocyte development during embryogenesis. However, the results obtained with the *promE-Gal4* driver, which becomes active at the L1 stage, indicate that larval oenocytes still remotely control the tracheal watertightness. Interestingly, the anterior spiracles become functional at the L2 larval stage [62]. Thus, that the air-filling phenotype does not occur earlier than the late L2 larval stage might be a sole result of the activity of the anterior spiracles.

In summary, we have discovered a novel VLCFA-dependent remote signal, which emanates from the oenocytes to prevent liquid accumulation in the respiratory system (Figure 7). Although the spiracles of *Drosophila* larvae develop at the posterior and anterior ends [16], their watertightness might depend on an evolutionarily conserved function of the oenocytes, given that spiracles and oenocytes are tightly connected in several insects species that contain metameric abdominal spiracles [15]. Finally, the discovery of this unexpected VLCFA-dependent function suggests that FA metabolic pathways mediate other unknown regulatory processes that must be systematically investigated.

## Materials and Methods

### Fly stocks and genetics

The following fly strains were used: *svp-Gal80* and *BO-Gal4* [14]; *Cg-Gal4* [63]; *daughterless(da)-Gal4*; *UAS-grim* [64]; *PBac[5HPw^+^]CG11198^B131^*, *FRT-42D,[PUbi-GFP]*, *actin5C>CD2>Gal4,UAS-GFP*, *UAS-Dcr-2*, (Bloomington Stock Center); Inducible RNA-interfering (*UAS-RNAi*) lines to *ACC*, *FAS^CG3523^*, *FAS^CG3524^*, *KAR*, *CG6660*, *LPR1*, *LPR2* were obtained from either VDRC or NIG-FLY. The P-element insertions (Exelixis collection) *PBac[WH]LpR2^f01682^* and *P[XP]^d11715^*, flanking the LpR2 gene and the *PBac[WH]LpR2^f03030^*, *P[XP]^d10508^*, flanking the LpR1 gene were used to generate deficiency as described [65].

All the UAS/Gal4 tests were performed with and without a *UAS-Dcr-2* that strengthens the RNAi effect [66]. Using the *UAS-Dcr-2* provided similar results with *Cg-Gal4* and *BO-Gal* drivers, except that the semi-lethal phenotype of *BO>KAR-RNAi* animals (Table 1, Line 12) was visible only after adding the *UAS-Dcr-2*. In addition, the phenotype induced by the *prom-Gal4* driver could be observed only after adding the *UAS-Dcr-2* and when performing the assay at 29°C to strengthen UAS/Gal4 activity.

The *ACC^oeTD^* (*ACC^B131^/ACC^B131^;da-Gal4,svp-Gal80/UAS-ACC*) mutant was produced by crossing *ACC^B131^/+;UAS-ACC/+* females to *ACC^B131^/+;da-Gal4,svp-Gal80/+* males. Since both lines were balanced by a co-segregating *SM5-TM6B,Tb^−^* balancer, the *ACC^oeTD^* mutant animals were identified by their *Tb^+^* phenotype. Since all the flies used contained P-w[+mC] transposable elements, the experiments were done in a *white* mutant (*w^−^*) background.

### Molecular biology

To generate the *UAS-ACC* transgenic line, the *ACC* (GH12002) cDNA was cloned into the pUAST vector using standard molecular biology techniques. The second exon of *FAS^CG17374^* and the corresponding reverse fragments were cloned into the pWIZ vector by SpeI-AvrII and NheI-XbaI respectively [67]. Plasmid constructs were injected by BestGene.

Quantitative PCR was performed as described [68] using RNA TRIzol (Invitrogen) to extract RNAs from 20 larvae for each sample. cDNAs were synthesized with SuperScript II and PCR was perfomed with a LightCycler 480 SYBR Green I Master (Roche Applied Science). Primers for *Charybdis* (*5′- CGACAACTTGGACGATG-3′* and *5′-CTCTCGAACTCAATGAAGAGC-3′*) and *Scylla* (*5′-GCAAGAAGACAAAGCCC-3′* and *5′- ACACTTCCGTACAGGT-3′*) were used and the mRNA amounts were normalized to *RpL32* (*5′-GACGCTTCAAGGGACAGTATCTG-3′* and *5′-AAACGCGGTTCTGCATGAG-3′*) mRNA values.

### Tissue analyses

The antiserum to ACC was produced after rabbit immunization with the peptides ^1746^EFSKARPTVDIRTPD^1760^ and ^2468^AQMQLNEETSNSNQG^2482^ (residue numbered according to the ACC-PA) and affinity purified by Eurogentec. Larval tissues were dissected in PBS, fixed in 4% paraformaldehyde for 20 min at room temperature on a rocking tray, rinsed twice in PBS 0.1% triton ×100 and blocked for 30 min in PBS-0.2%BSA-0.1% triton ×100. For antibody staining, the ACC antiserum or a mouse GFP antibody (Roche) were used at 1∶1000 and 1∶200 dilutions respectively and incubated overnight at 4°C. After extensive washes, tissues were incubated with secondary goat anti-rabbit-A568 or rabbit anti-mouse-A488 (Invitrogen) at a 1∶500 dilution for 2 h at room temperature. Nuclear labeling was performed by incubating the tissues with a 0.01 mM solution of TO-PRO-3-iodide (Invitrogen) in DABCO (Sigma) for at least 2 days at 4°C. For lipid detection, the tissues were incubated for 45 minutes with either a filtrated 0.1% Oil-Red-O solution [14] or a Nile Red dilution [69]. Tissues were mounted separately and analyzed on a fluorescent Leica (Leitz DMRB) or a confocal Nikon (TE 2000-U) microscope.

### Animal analyses

To determine larval lethality, flies were allowed to lay eggs on a grape-juice plate for 4 h and kept at 25°C for embryonic development. The next day, newly hatched larvae were collected over a 5 h period and groups of 50 larvae were transferred onto agar plates containing a small piece of food. The dead and living larvae were counted twice per day. A similar experimental protocol was used with 70-hour-old larvae to determine the percentage of animals that stray away from food; the larvae out of the food were counted 1 h after larval transfer. Tracheal air filling was observed with a Leica microscope (Leitz DMRB) on living larvae after cold-induced immobilization.

### “Water-tightness” assay

Larvae were collected and transferred into tinted food containing 1% brilliant blue FCF (referred to as E133 in food industry) in the middle of agar plates. Wild-type larvae at either the L2 or L3 stages were analyzed at various times over a 36 h period. Mutant larvae without visible tracheal or behavioral phenotypes were transferred into the tinted food and analyzed once they strayed away from food. Larvae were mounted in 80% glycerol, immobilized by cold and observed with a Leica MZFLIII dissecting microscope.

### Metabolic measurements

To evaluate metabolic changes in post-feeding larvae, animal were grown in controlled density on standard medium. 0–4 hour prepupae were collected and analyzed as described [70] using a TRIGLYCERIDE LIQUICOLR kit (STANBIO Laboratory) for total TG levels, a Protein Assay reagent (Bio-Rad) for total protein levels, and a GLUCOSE (GOD-POD) kit (Thermo Electron) for glucose levels. Trehalose and glycogen were evaluated by measuring the glucose produced after overnight enzymatic digestion with either Trehalase [70] or amyloglucosidase [71], respectively. A Denver APX-60 scale was used to weigh female prepupae; the same results were obtained with male prepupae (data not shown). NEFA levels were evaluated after extraction of 100 mg of 0–4 h prepupae and analyzed as previously described [72]. To determine TG consumption, eggs were collected on grape-juice/agar plates for 4 h; the following day, newly-hatched larvae were collected over a 5 h period and transferred on standard media. At the given developmental times, groups of 40 (19–24 h after hatching) or 30 (40–45 h after hatching) larvae of roughly the same size were collected and either directly frozen or starved for 14 h in PBS. The TG contents were measured as described above.

## Supporting Information

Figure S1 Sequence comparison of ACC from *Drosophila* and mouse. Sequence alignment of the *Drosophila* ACC-PB (Dm) and of the mouse ACC1 (Mm) polypeptides. Comparison of the proteins reveals an overall identity of 62% and a similarity of 77%. The biotin carboxylase (BC, light grey), the biotin carboxyl carrier protein (BCCP, underlined), and the acetyl-CoA carboxytransferase (CT, dark grey) domains are conserved. The lysine residue (bold) that covalently links the biotin is conserved.
(PDF)

Figure S2 The ACC Locus. Introns (lines) and exons (boxes) of the *Drosophila ACC* gene. The *ACC* gene is oriented according to flybase; the polypeptide (colored boxes) contains, from right to left, a BC and a BCCP domain in the N-terminal moiety (black boxes) and an acetyl-CoA carboxytransferase domain (dark grey boxes) in the C-terminal moiety. The insertion site of the *ACC^B131^* P-element is indicated (arrow).
(TIF)

Figure S3 ACC expression in flip-out clones expressing *ACC-RNAi*. Direct GFP fluorescence (A,D,G), staining to ACC (B,E,H) and nuclei (B,E), and merge pictures (C,F,I) in fat body (A–C), oenocytes (D–F) and imaginal disc (G–I). Note that the ACC signal is severely suppressed in GFP-positive cells that co-express the *ACC-RNAi*. Scale bars: 20 µm.
(TIF)

Figure S4 Oil-Red-O detection of LDs in oenocytes. (A) Accumulation of LDs in *ACC-RNAi* flip-out oenocyte (arrows). (B) Lack of fast-induced accumulation of LDs in *LpR2-RNAi* flip-out oenocytes (arrows). (C) Lack of LD accumulation in *ACC-RNAi*, *LpR2-RNAi* flip-out oenocytes (arrows). (D) Lack of LD accumulation in an *ACC^B131^* homozygote oenocyte (arrow) generated in an *LpR2* mutant background.
(TIF)

Figure S5 Loci of LpR1 and LpR2. Introns (lines) and exons (boxes) of the *Drosophila LpR2* and *LpR1* genes located in tandem on the third chromosome. According to flybase, both genes are oriented from right to left. The coding sequences are indicated (grey boxes). The insertions points of the *PBac[WH]LpR2^f01682^*, *P[XP]^d11715^*, *PBac[WH]LpR2^f03030^* and *P[XP]^d10508^* are indicted (arrows). The FRT-recombination between two P-elements removed the genomic sequences located between their insertion sites (arrows) [65]. Recombination between The insertions point of the *PBac[WH]LpR2^f01682^* and *P[XP]^d11715^*, and between *PBac[WH]LpR2^f03030^* and *P[XP]^d10508^* (Exelixis collection) produced deficiencies of *LpR2* and *LpR1*, respectively.
(TIF)

Figure S6 Tracheal phenotype of control and mutant larvae. Visualization of the tracheal system of larvae of the following genotypes, from top to bottom: *BO>+* control L2 larva *BO>grim* L2 larva *BO>ACC-RNAi* L2 larva *BO>+* control L3 larva *BO;svp-Gal80>ACC-RNAi* L3 larva *BO>FAS^CG17374^-RNAi* L3 larva *BO>CG6660-RNAi* L3 larva *promE>grim* L3 larva *promE>ACC-RNAi* L3 larva *promE>FAS^CG17374^-RNAi* L3 larva *promE>CG6660-RNAi* L3 larva
(TIF)

Figure S7 Hypoxic response and TG consumption in L1 and L2 larvae. (A–B) Expression of the hypoxic-induced genes *charybdis* and *scylla* in late L1 (A) and mid/late L2 (B) larvae of the following genotypes: (*BO*) *BO* control; (*ACC-Ri*) *BO>ACC-RNAi*, (*grim*) *BO>grim*. The hypoxic responsive genes are significantly induced in mid/late L2 larvae (B) but not in late L1 larvae (A). (A′–B′) TG consumption in late L1 (A′) and mid/late L2 (B′) larvae before (white bars) and after overnight starvation (black bars). T test: *: P<0.05; **: P<0.01; ***: P<0.001.
(TIF)
