# Supplementary material for: Drosophila melanogaster Acetyl-CoA-Carboxylase Sustains a Fatty Acid–Dependent Remote Signal to Waterproof the Respiratory System
Source: PLoS Genet. 2012 Aug 30;8(8):e1002925. doi: 10.1371/journal.pgen.1002925 (PMC3431307; doi:10.1371/journal.pgen.1002925)
Supplement: Figure S1 — Sequence comparison of ACC from Drosophila and mouse. Sequence alignment of the Drosophila ACC-PB (Dm) and of the mouse ACC1 (Mm) polypeptides. Comparison of the proteins reveals an overall identity of 62% and a similarity of 77%. The biotin carboxylase (BC, light grey), the biotin carboxyl carrier protein (BCCP, underlined), and the acetyl-CoA carboxytransferase (CT, dark grey) domains are conserved. The lysine residue (bold) that covalently links the biotin is conserved. (PDF) [file pgen.1002925.s001.pdf]

```
Dm ------------MSETNESNDTAAQSAEGERPSFLVGDEIDERAAEAGEACDEFPLKMQNDVRQNGDISERRKRLRPSMSRGTGLGQDRHQ---DRDFHIATTEEFVKRFGGTRVINKV
                ...     .....   .. : ::  ...:. .  . :          .  .    :    . :   :  . .  :.::..   .::: .:.  ::: ::::..::.::
Mm MDEPSPLAKTLELNQHSRFIIGSVSEDNSEDEISNLVKLDLEEKEGSLSPASVSSDTLSDLGISGLQDGLAFHMRSSMSGLHLVKQGRDRKKIDSQRDFTVASPAEFVTRFGGNKVIEKV

Dm LIANNGIAAVKCMRSIRRWAYEMFKNERAIRFVVMVTPEDLKANAEYIKMADHYVPVPGGSNNNNYANVELIVDIALRTQVQAVWAGWGHASENPKLPELLHKEGLVFLGPPERAMWALG
   ::::::::::::::::::.::::.::::::::::::::::::::::::::::::::::: ::::::::::.::: :  ::::::::::::::::::::: :.:..:.::: .::::::
Mm LIANNGIAAVKCMRSIRRWSYEMFRNERAIRFVVMVTPEDLKANAEYIKMADHYVPVPGGPNNNNYANVELILDIAKRIPVQAVWAGWGHASENPKLPELLLKNGIAFMGPPSQAMWALG

Dm DKVASSIVAQTAEIPTLPWSGSDLKAQ-----YSGKKIKISSELFARGCVTNVEQGLAAVNKIGFPVMIKASEGGGGKGIRRVDTTEEFPGLFRQVQAEVPGSPIFVMKLARGARHLEVQ
   ::.::::::::: :::::::: :...     .: . ... ..:. .: : .:..:: :....:.:::::::::::::::::.:.....::.::::::::::::::::::.::. .::::::
Mm DKIASSIVAQTAGIPTLPWSGSGLRVDWQENDFSKRILNVPQDLYEKGYVKDVDDGLKAAEEVGYPVMIKASEGGGGKGIRKVNNADDFPNLFRQVQAEVPGSPIFVMRLAKQSRHLEVQ

Dm LLADQYGNAISLFGRDCSIQRRHQKIIEEAPAIVAQPEVFEDMEKAAVRLAKMVGYVSAGTVEYLYDPEGRYFFLELNPRLQVEHPCTEMVADVNLPAAQLQIGMGIPLYRLKDIRLLYG
   .::::::::::::::::.:::::::::::: .: : ::: ::. ::.::::::::::::::::::. .: ..:::::::::::::::::::::::::::::::::.:::::.:.::::..::
Mm ILADQYGNAISLFGRDCSVQRRHQKIIEEAPAAIATPAVFEHMEQCAVKLAKMVGYVSAGTVEYLYSQDGSFYFLELNPRLQVEHPCTEMVADVNLPAAQLQIAMGIPLFRIKDIRMMYG

Dm ESPWGSSVIDFENPPNKPRPSGHVIAARITSENPDEGFKPSSGTVQELNFRSSKNVWGYFSVAASGGLHEFADSQFGHCFSWGENRQQARENLVIALKELSIRGDFRTTVEYLITLLETN
    ::::.. :::::  . : : ::::::::::::::::::::::::::::::::.:::::::::::.:::::::::::::::::::::::::..:  :.:.:::::::::::::::::::: ::::.
Mm VSPWGDAPIDFENSAHVPCPRGHVIAARITSENPDEGFKPSSGTVQELNFRSNKNVWGYFSVAAAGGLHEFADSQFGHCFSWGENREEAISNMVVALKELSIRGDFRTTVEYLIKLLETE

Dm RFLDNSIDTAWLDALIAERVQSEKPDILLGVMCGSLHIADRQITESFSSFQTSLEKGQIQAANTLTNVVDVELINDGIRYKVQAAKSGANSYFLLMNSSFKEIEVHRLSDGGLLISLEGA
    :  : :::.::: ::::.::.:.:: .:::.::.::.:: .. .:.:.:  :::.::.  :.:: :.::::: .::.: ....... ::: ..::.:  :..:::::::::.: .:.
Mm SFQLNRIDTGWLDRLIAEKVQAERPDTMLGVVCGALHVADVSLRNSISNFLHSLERGQVLPAHTLLNTVDVELIYEGIKYVLKVTRQSPNSYVVIMNGSCVEVDVHRLSDGGLLLSYDGS

Dm SYTTYMKEEVDRYRIVIGNQTCVFEKENDPSLLRSPSAGKLINMIVEDGAHVSKGQAYAEIEVMKMVMTLTSQEAGTVTFVRRPGAVLDAGSLLGHLELDDPSLVTKAQPFKGQFLQPEN
   ::::::::::::::.:::.::::::::::::.:::::::::::.:::::.::  :: ::::::::::::::. ::: . .:.::::.:: : ......::.:: : .:.   :.. : ..
Mm SYTTYMKEEVDRYRITIGNKTCVFEKENDPSVMRSPSAGKLIQYIVEDGGHVFAGQCYAEIEVMKMVMTLTAVESGCIHYVKRPGAALDPGCVIAKMQLDNPSKVQQAELHTGSLPQIQS

Dm APV-PEKLNRVHNTYKSILENTLAGYCLPEPFNAQRLRDIIEKFMQSLRDPSLPLLELQEVIASISGRIPISVEKKIRKLMTLYERNITSVLAQFPSQQIASVIDSHAATLQKRADRDVF
   . .   :::.:: .    . : :.. :::::.:: ..:..: .:..:.:::::::::::::....:.:::::..:::.:.: :. :   :::::: ::::::::..:::::::.....:.::
Mm TALRGEKLHRVFHYVLDNLVNVMNGYCLPDPFFSSRVKDWVERLMKTLRDPSLPLLELQDIMTSVSGRIPLNVEKSIKKEMAQYASNITSVLCQFPSQQIANILDSHAATLNRKSEREVF

Dm FLTTQSIVQLVQRYRNGIRGRMKAAVHELLRQYYDVESQFQYGHYDKCVGLVREHNKDDMQTVVNTIFSHSQVAKKNLLVTLLIDHLWANEPGLTDELANTLSELTSLNRAEHSRVALRS
   :..::::::::::::.::::.::::.: .:::::  ::.::: :::::::   .::.::.::.::.: ::::.::.:::::::.::::.: . .: ::::: : :.:::.:.. ...:::::.
Mm FMNTQSIVQLVQRYRSGIRGHMKAVVMDLLRQYLRVETQFQNGHYDKCVFALREENKSDMNTVLNYIFSHAQVTKKNLLVTMLIDQLCGRDPTLTDELLNILTELTQLSKTTNAKVALRA

Dm RQVLIAAHQPAYELRHNQMESIFLSAVDMYGHDFHPENLQRLILSETSIFDILHDFFYHSNRAVCNAALEVYVRRAYTSYELTCLQHLELSGGLPLVHFQFLLPTAHPNRLFSRMSSPDG
   ::::::.: :.:::::::.::::::::.::::::.:  ::::.::::::::::.: .:::::..:  :::::::::: .:::. .:: .:. .  .:.:::.:::.::::      ..
Mm RQVLIASHLPSYELRHNQVESIFLSAIDMYGHQFCIENLQKLILSETSIFDVLPNFFYHSNQVVRMAALEVYVRRAYIAYELNSVQHRQLKDNTCVVEFQFMLPTSHPNR-----GNIPT

Dm LDQAAAESLGNSFVRTGAIAAFDSFEHFEMYSDEILDLLEDFVSPAMVNAKVLEAVEAADSISDSRHSTSINVSLSDPVTRANAAEEAKSTEPIHIVSVAVRETGELDDLQMAQIFGNYC
   :.. .  :  : .  : . .. : .  ..          . .  .::     .  :    :    . ::  ..       .   :      ...   ::::..::..  :...: ..: .: ..
Mm LNRMSFASNLNHYGMTHVASVSDVL--LDNAFTPPCQRMGGMVSFRTFEDFVRIFDEIMGCFCDSPPQSPTFPESGH--TSLYDEDKVPRDEPIHILNVAIKTDGDIEDDRLAAMFREFT

Dm QEHNEELFQRRIRRITFAALKK---------------RQFPKFFTFRARDKFTEDRIYRHLEPASAFHLELNRMKTYDLEALPTANQKMHLYLGKAKVSKGQEVTDYRFFIRSIIRHSDL
   :...  : .. :::.:: . .:                :.:::::::::::: ::::::::: ::.::::::...:: :.: ::.:::::: :::  : :::::::.:.:::::::
Mm QQNKATLVEHGIRRLTFLVAQKDFRKQVNCEVDQRFHREFPKFFTFRARDKFEEDRIYRHLEPALAFQLELNRMRNFDLTAIPCANHKMHLYLGAAKVEVGTEVTDYRFFVRAIIRHSDL

Dm ITKEASFEYLQNEGERVLLEAMDELEVAFSHPHAKRTDCNHIFLNFVPTVIMDPAKIEESVTKMIMRYGPRLWKLRVLQAELKMVIRQSPQSPTQAVRLCIANDSGYFLDISMYTEQTEP
   .:::::::::::::::.:::::::::::::.. .. ::::::::::::::::::::.:::::: .:.:::: ::::::::::::. :: .   . .  .:: ..:.:::.::::.: : :.
Mm VTKEASFEYLQNEGERLLLEAMDELEVAFNNTNV-RTDCNHIFLNFVPTVIMDPSKIEESVRSMVMRYGSRLWKLRVLQAELKINIRLTTTGKAIPIRLFLTNESGYYLDISLYKEVTDS

Dm ETGIIKFKAYGEKQGSLHGHPISTPYMTKDFLQQKRFQAQSNGTTYVYDVPDMFRQMTERHWREFSKARPTVDIRTPDKILIECKELVLEGDNLVEMQRLPGENNCGMVAWRIVLATPEY
   .:. : :.:::.::: :::  :.:::.:::.::.::::::: ::::.::.:.::::     . :. .:        .      :. ::     . .. .   .::.:.:::: :. :::::.. : .:::
Mm RTAQIMFQAYGDKQGPLHGMLINTPYVTKDLLQSKRFQAQSLGTTYIYDIPEMFRQSLIKLWESMSTQAFLPSPPLPSDILTYTELVLDDQGQLVHMNRLPGGNEIGMVAWKMSLKSPEY

Dm PNGREIIVIANDLTYLIGSFGIKEDVLFAKASQLARQLKVPRIYISVNSGARIGLAEEVKAMFKIAWEDPEEPDKGFKYLYLSTEDYAQVANLNSVRAILIEDEGEQRYKITDIIGKDDG
   :.::.::::.::.:: ::::: .::.:: .::.:::   .:::::..:::::::::::.. ::..:: :::.: ::.:::::. .:: .:. ::::.   .::::::.::::::::::..:
Mm PDGRDIIVIGNDITYRIGSFGPQEDLLFLRASELARAEGIPRIYVAANSGARIGLAEEIRHMFHVAWVDPEDPYKGYKYLYLTPQDYKRVSALNSVHCEHVEDEGESRYKITDIIGKEEG

Dm LGVENLRYAGLIAGETSQAYEEIVTIAMVTCRTIGIGSYVVRLGQRVIQIDNSHIILTGYAALNKLLGRKVYASNNQLGGTQIMFNNGVTHKTEAIDLDGVYTILDWLSYIPAYIGCDLP
   ::.:::: .:.:::::.: ::.:..::..::::.::::.:::::::.::..:::.:::: .::::.:::.::.::::::: ::: :::::.:   :..::.:.: ::::.:    .   ..:
Mm LGAENLRGSGMIAGESSLAYDEVITISLVTCRAIGIGAYLVRLGQRTIQVENSHLILTGAGALNKVLGREVYTSNNQLGGIQIMHNNGVTHSTVCDDFEGVFTVLHWLSYMPKSVHSSVP

Dm IVLPNDRIERPVDFMPTKSPYDPRWMLGGRVNPVNANDWENGFFDRDSWSEIMASWAKTVVTGRARLGGVPVGVIAVETRTVEVEMPADPANLDSEAKTLQQAGQVWYPDSSYKTAQAIK
   ..   .: :.: ..:.:::.::::::::.:: .:.. ..: .:::: :.:::: ::.:::.::::::.:::.::::::::. .::::::::::: .:::::::.:::..:: ::::
Mm LLNSKDPIDRIIEFVPTKAPYDPRWMLAGRPHPTQKGQWLSGFFDYGSFSEIMQPWAQTVVVGRARLGGIPVGVVAVETRTVELSIPADPANLDSEAKIIQQAGQVWFPDSAFKTYQAIK

Dm DFGREELPLIVFANWRGFSGGMKDMYEQIVKFGAYIVDGLREYKKPVLIYLPPNAELRGGAWAVLDSLINPRYMETYADPEARGGVLEPEGIVEIKYKEKDLVKTIHRLDPTTIALKKEL
   ::.:: :::.:::::::::::::::.:..::::::::::: ..::..:.::.::::::.:.:.:  ::::.:: ::: :.::.::::: ::::...::::::..:.::. : : ..:
Mm DFNREGLPLMVFANWRGFSGGMKDMYDQVLKFGAYIVDGLRECSQPVMVYIPPQAELRGGSWVVIDPTINPRHMEMYADRESRGSVLEPEGTVEIKFRKKDLVKTMRRVDPVYIRLAERL

Dm DEANASGDKVRAAQVDEKIKARIAVLMHVYHTVAVHFADLHDTPERMLEKECISEIVPWRDSRRWLYWRLRRLLLEDAYIKKILRAQDNLSVGQAKQMLRRWLVEEKGATEAYLWDKNEE
     . :  . .  ... :.: :   :. .:: :::.:::::::: :: ::  :..:. :. :: ..:::::::::::   :::  :. .:. :: . ::::.:: .:...::.::.:..
Mm GTPELSPTERK--ELESKLKEREEFLIPIYHQVAVQFADLHDTPGRMQEKGVINDILDWKTSRTFFYWRLRRLLLEDLVKKKIHNANPELTDGQIQAMLRRWFVEVEGTVKAYVWDNNKD

Dm MVSWYEEQINAE----SIVSRNVNSVRRDAIISTISKMLEDCPDVALDAVVGLCQGLTPVNRGVVVRTLAQMQLNEETSNSNQG
   .: : :.:.. :    :.. .:.. . :: ... : ....  :.::.:..: . : ..:..:. ::: :. :.
Mm LVEWLEKQLTEEDGVRSVIEENIKYISRDYVLKQIRSLVQANPEVAMDSIVHMTQHISPTQRAEVVRILSTMDSPST-------
```

Parvy et al., Figure S1
