# Supplementary figures and images for: Drosophila melanogaster Acetyl-CoA-Carboxylase Sustains a Fatty Acid–Dependent Remote Signal to Waterproof the Respiratory System
Source: PLoS Genet. 2012 Aug 30;8(8):e1002925. doi: 10.1371/journal.pgen.1002925 (PMC3431307; doi:10.1371/journal.pgen.1002925)

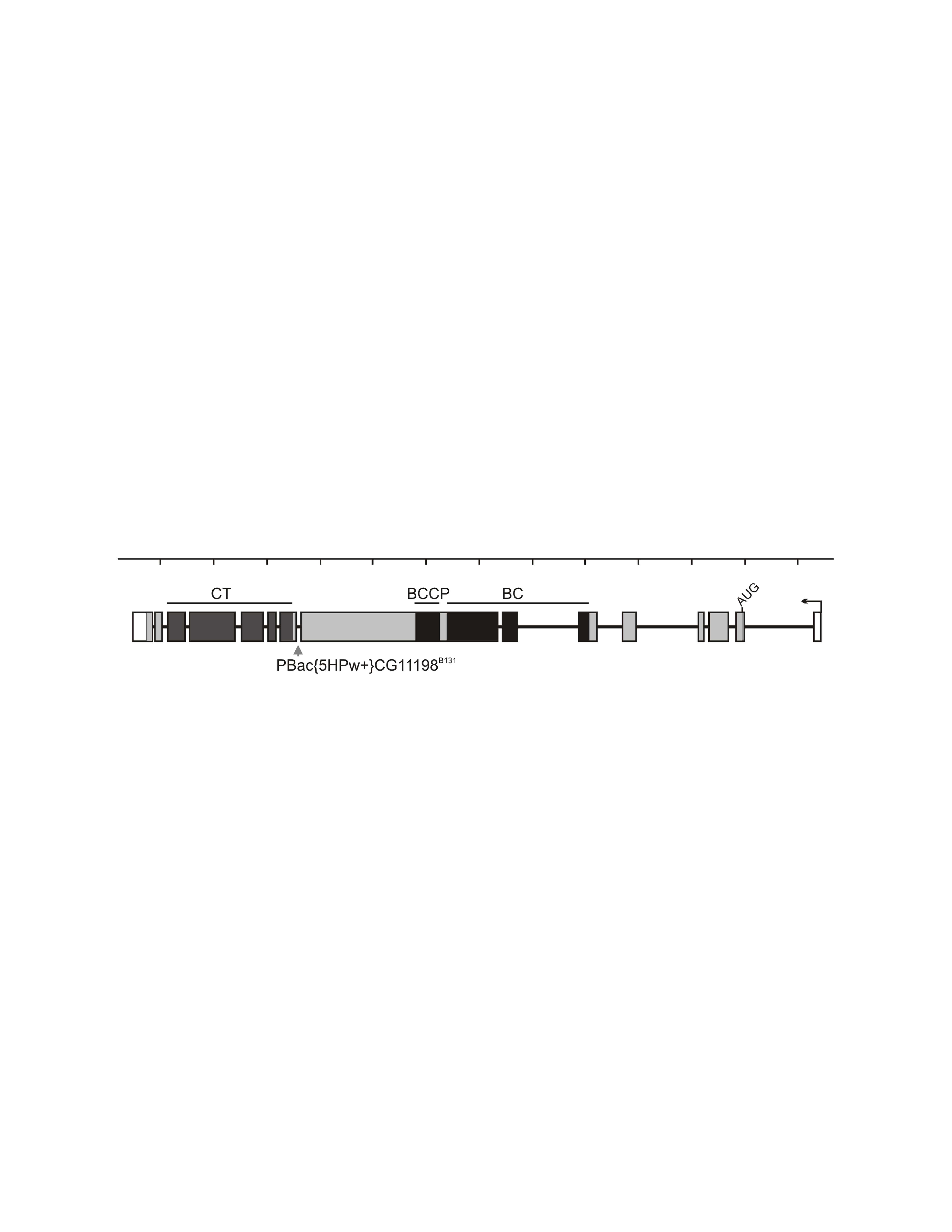

Supplement: Figure S2 — The ACC Locus. Introns (lines) and exons (boxes) of the Drosophila ACC gene. The ACC gene is oriented according to flybase; the polypeptide (colored boxes) contains, from right to left, a BC and a BCCP domain in the N-terminal moiety (black boxes) and an acetyl-CoA carboxytransferase domain (dark grey boxes) in the C-terminal moiety. The insertion site of the ACCB131 P-element is indicated (arrow). (TIF) [file pgen.1002925.s002.tif]

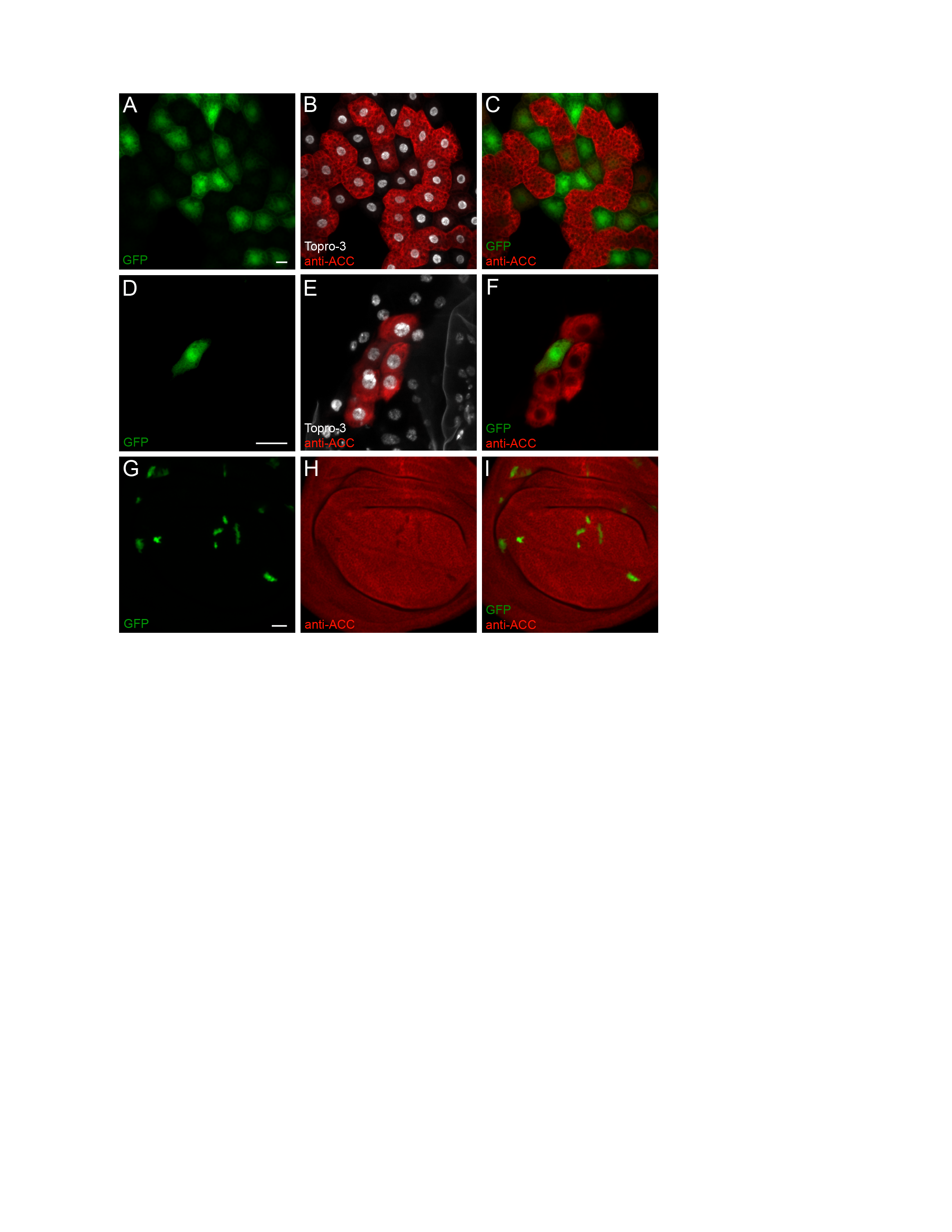

Supplement: Figure S3 — ACC expression in flip-out clones expressing ACC-RNAi. Direct GFP fluorescence (A,D,G), staining to ACC (B,E,H) and nuclei (B,E), and merge pictures (C,F,I) in fat body (A–C), oenocytes (D–F) and imaginal disc (G–I). Note that the ACC signal is severely suppressed in GFP-positive cells that co-express the ACC-RNAi. Scale bars: 20 µm. (TIF) [file pgen.1002925.s003.tif]

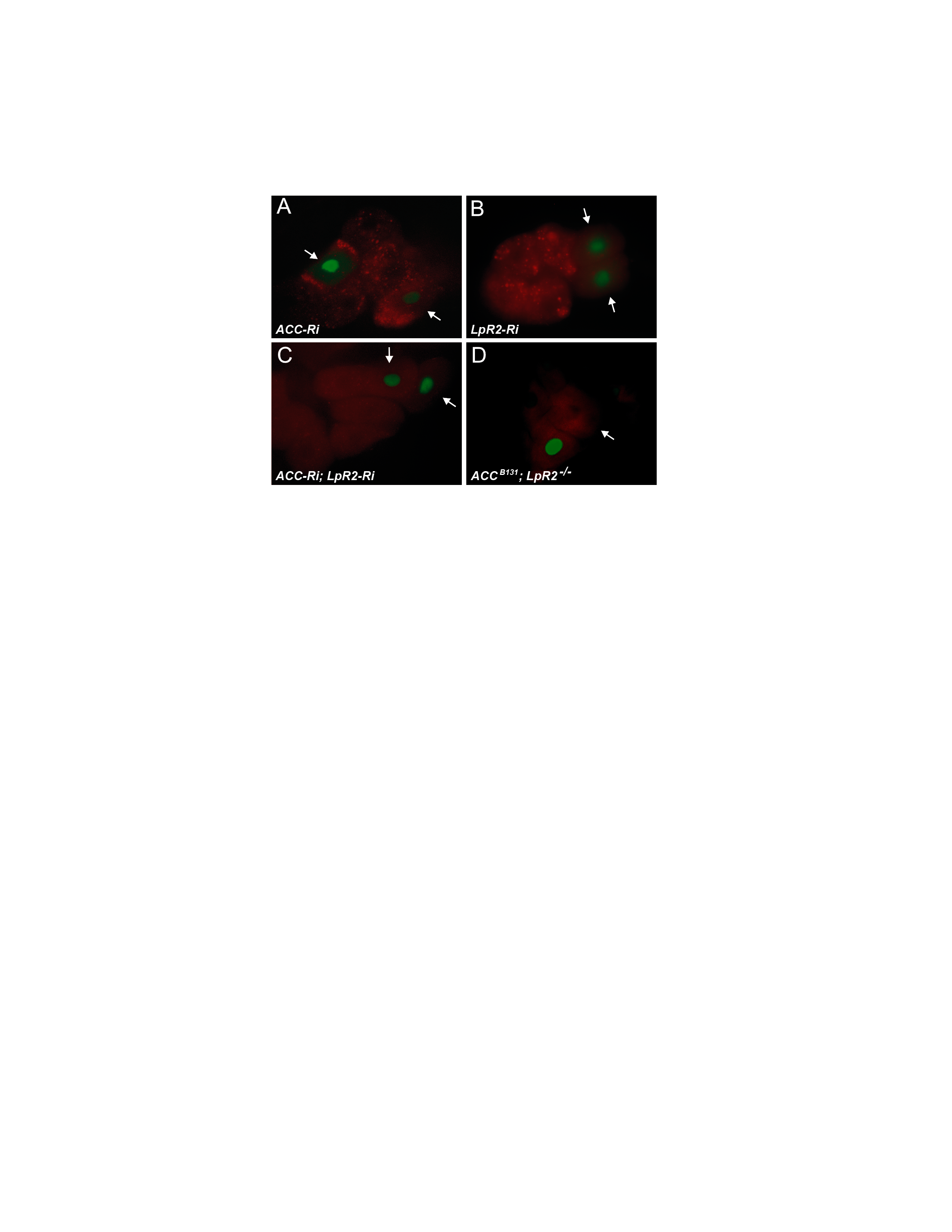

Supplement: Figure S4 — Oil-Red-O detection of LDs in oenocytes. (A) Accumulation of LDs in ACC-RNAi flip-out oenocyte (arrows). (B) Lack of fast-induced accumulation of LDs in LpR2-RNAi flip-out oenocytes (arrows). (C) Lack of LD accumulation in ACC-RNAi, LpR2-RNAi flip-out oenocytes (arrows). (D) Lack of LD accumulation in an ACCB131 homozygote oenocyte (arrow) generated in an LpR2 mutant background. (TIF) [file pgen.1002925.s004.tif]

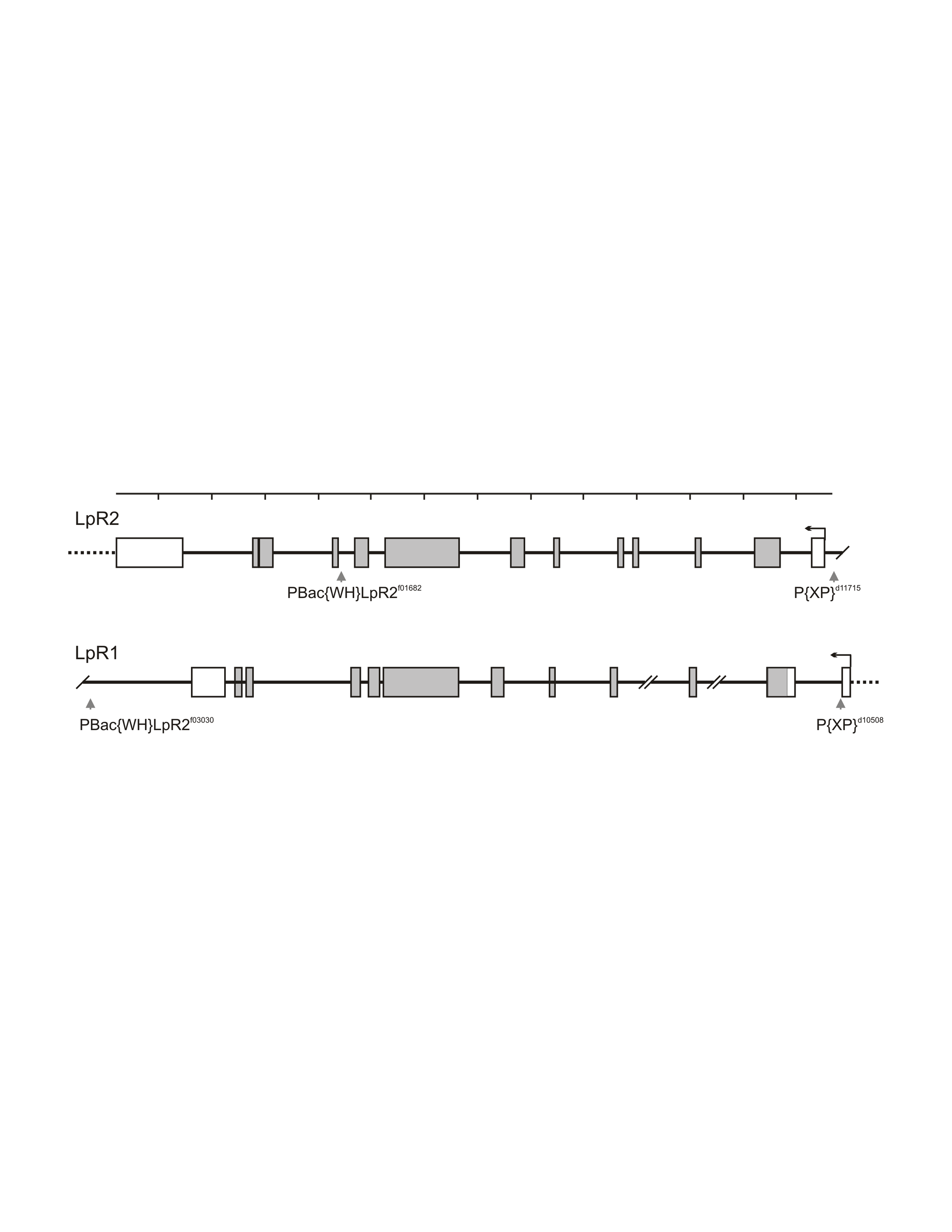

Supplement: Figure S5 — Loci of LpR1 and LpR2. Introns (lines) and exons (boxes) of the Drosophila LpR2 and LpR1 genes located in tandem on the third chromosome. According to flybase, both genes are oriented from right to left. The coding sequences are indicated (grey boxes). The insertions points of the PBac[WH]LpR2f01682, P[XP]d11715, PBac[WH]LpR2f03030 and P[XP]d10508 are indicted (arrows). The FRT-recombination between two P-elements removed the genomic sequences located between their insertion sites (arrows) [65]. Recombination between The insertions point of the PBac[WH]LpR2f01682 and P[XP]d11715, and between PBac[WH]LpR2f03030 and P[XP]d10508 (Exelixis collection) produced deficiencies of LpR2 and LpR1, respectively. (TIF) [file pgen.1002925.s005.tif]

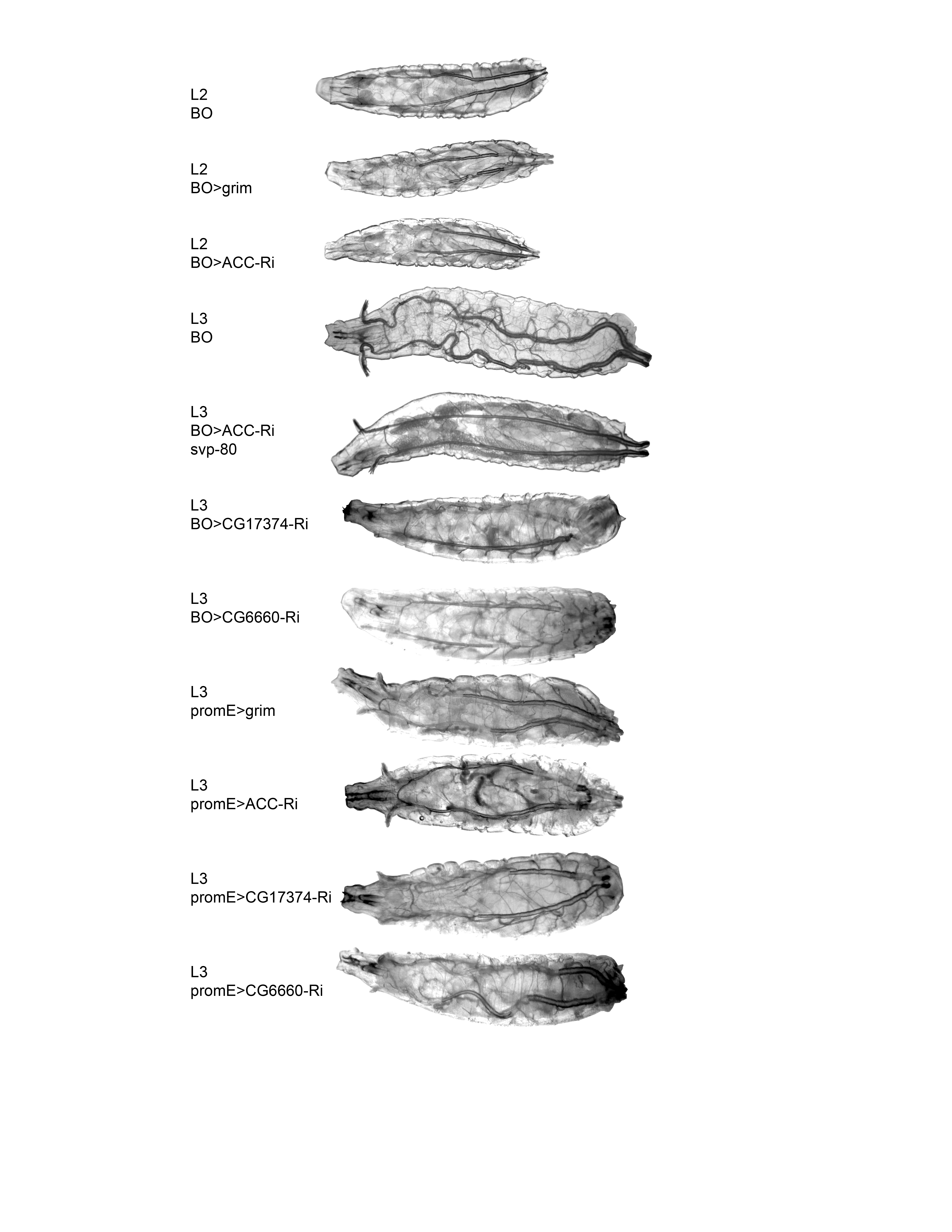

Supplement: Figure S6 — Tracheal phenotype of control and mutant larvae. Visualization of the tracheal system of larvae of the following genotypes, from top to bottom: BO>+ control L2 larva BO>grim L2 larva BO>ACC-RNAi L2 larva BO>+ control L3 larva BO;svp-Gal80>ACC-RNAi L3 larva BO>FASCG17374-RNAi L3 larva BO>CG6660-RNAi L3 larva promE>grim L3 larva promE>ACC-RNAi L3 larva promE>FASCG17374-RNAi L3 larva promE>CG6660-RNAi L3 larva (TIF) [file pgen.1002925.s006.tif]

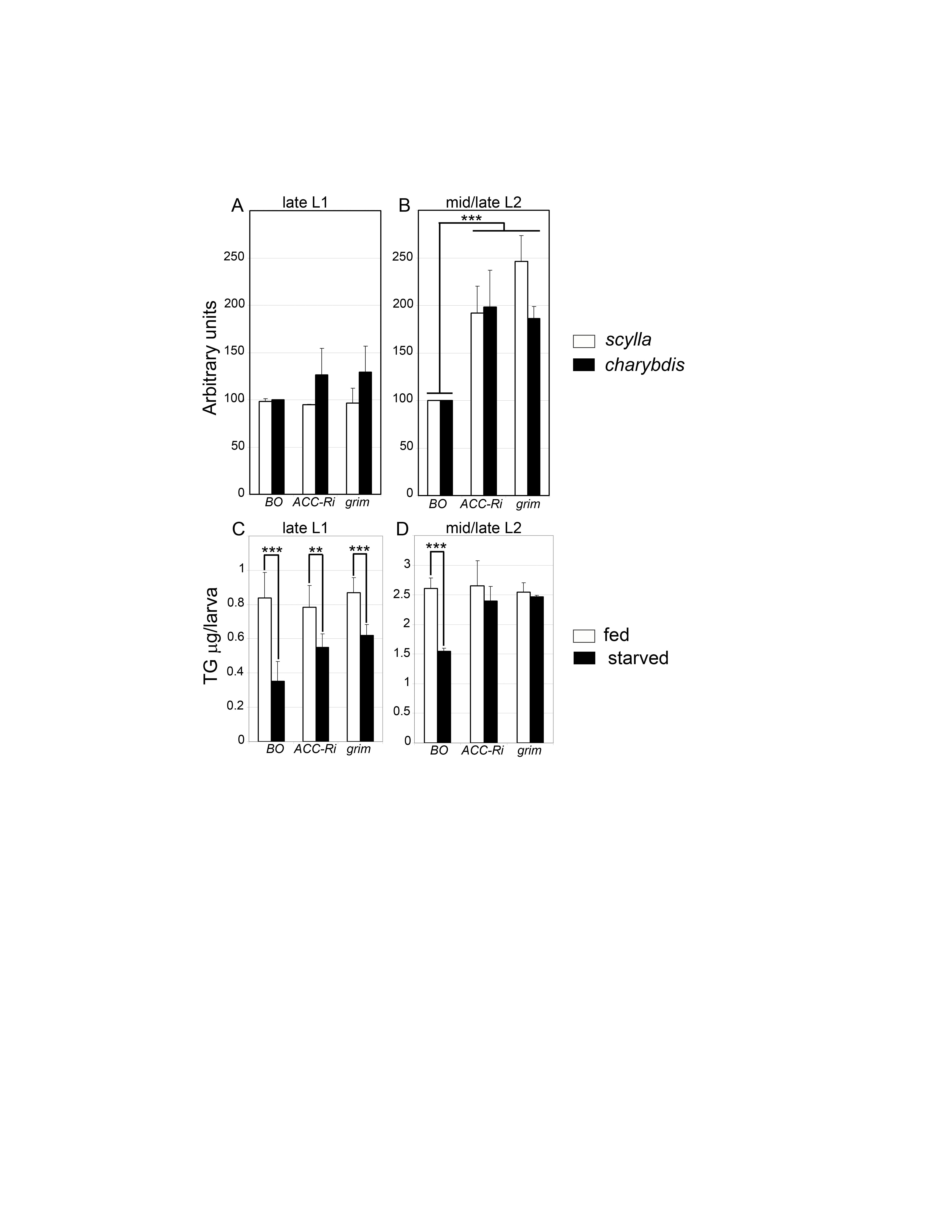

Supplement: Figure S7 — Hypoxic response and TG consumption in L1 and L2 larvae. (A–B) Expression of the hypoxic-induced genes charybdis and scylla in late L1 (A) and mid/late L2 (B) larvae of the following genotypes: (BO) BO control; (ACC-Ri) BO>ACC-RNAi, (grim) BO>grim. The hypoxic responsive genes are significantly induced in mid/late L2 larvae (B) but not in late L1 larvae (A). (A′–B′) TG consumption in late L1 (A′) and mid/late L2 (B′) larvae before (white bars) and after overnight starvation (black bars). T test: *: P<0.05; **: P<0.01; ***: P<0.001. (TIF) [file pgen.1002925.s007.tif]
